# Oral vaccination of mice with attenuated *Salmonella* encoding *Trichinella spiralis* calreticulin and serine protease 1.1 confers protective immunity in BALB/c mice

**DOI:** 10.1371/journal.pntd.0010929

**Published:** 2022-11-29

**Authors:** Sheng Jie Bai, Lu Lu Han, Ruo Dan Liu, Shao Rong Long, Xi Zhang, Jing Cui, Zhong Quan Wang

**Affiliations:** Department of Parasitology, Medical College, Zhengzhou University, Zhengzhou, People’s Repuplic of China; University of Utah, UNITED STATES

## Abstract

**Background:**

*Trichinella spiralis* is a foodborne parasitic nematode which is a serious risk to meat safety. Development of anti-*Trichinella* vaccine is needed to control *Trichinella* infection in food animals. In this study, two novel *T*. *spiralis* genes (calreticulin and serine protease 1.1) in combination were used to construct oral DNA vaccines, and their induced protective immunity was evaluated in a murine model.

**Methodology/Principal findings:**

TsCRT+TsSP1.1, TsCRT and TsSP1.1 DNA were transformed into attenuated *Salmonella typhimurium* ΔcyaSL1344. Oral vaccination of mice with TsCRT+TsSP1.1, TsCRT and TsSP1.1 DNA vaccines elicited a gut local mucosal sIgA response and systemic Th1/Th2 mixed response. Oral vaccination with TsCRT+TsSP1.1 induced obviously higher level of serum specific antibodies, mucosal sIgA and cellular immune response than either of single TsCRT or TsSP1.1 DNA vaccination. Oral vaccination of mice with TsCRT+TsSP1.1 exhibited a 53.4% reduction of enteral adult worms and a 46.05% reduction of muscle larvae, conferred a higher immune protection than either of individual TsCRT (44.28 and 42.46%) or TsSP1.1 DNA vaccine (35.43 and 29.29%) alone. Oral vaccination with TsCRT+TsSP1.1, TsCRT and TsSP1.1 also obviously ameliorated inflammation of intestinal mucosa and skeletal muscles of vaccinated mice after challenge.

**Conclusions:**

TsCRT and TsSP1.1 might be regarded the novel potential targets for anti-*Trichinella* vaccines. Attenuated *Salmonella*-delivered DNA vaccine provided a prospective approach to control *T*. *spiralis* infection in food animals.

## Author summary

In this study, the fusion gene TsCRT+TsSP1.1 was designed and synthesized, the fusion gene TsCRT+TsSP1.1, individual TsCRT or TsSP1.1 was used to construct the DNA vaccines. Oral vaccination with TsCRT+TsSP1.1 in combination elicited obviously higher level of serum specific antibody, mucosal sIgA and cellular response, and immune protection than either of single TsCRT or TsSP1.1 DNA vaccination. Moreover, vaccination with TsCRT+TsSP1.1, TsCRT and TsSP1.1 also obviously relieved intestinal and muscle inflammatory reaction in vaccinated mice after challenge. The results indicated that TsCRT and TsSP1.1 might be regarded as the novel potential targets for anti-*Trichinella* vaccines.

## Introduction

*Trichinella spiralis* is an intestinal and tissue parasitic nematode of the genus *Trichinella* with a broad range of hosts including over 150 kinds of animals around the world [1]. Human *T*. *spiralis* infection results from ingesting raw or undercooked animal meat contained with the encapsulated infectious muscle larvae (ML). Trichinellosis was mainly reported in developing countries of Eastern Europe, South America (Argentina and Chile) and Southeast Asia [2,3]. Pork from domestic pigs is the major source of human trichinellosis [4]. From 2009 to 2020, eight outbreaks with 479 cases and 2 deaths were documented in China, and seven outbreaks (87.50%) were resulted from eating raw or semi-cooked pork [5]. *Trichinella* infection in food animals is a serious threat to the meat food safety [6,7]. Therefore, it’s necessary to develop anti-*Trichinella* vaccines to interrupt *Trichinella* infection in domestic pigs, and eliminate the ML in pork [8,9]

After contaminated meat is eaten, *T*. *spiralis* ML are released from the capsules in stomach under the digestion of gastric juice, and activated into intestinal infectious larvae (IIL) after exposure to bile [10,11]. The IIL intrude into gut epithelium, and develop to adult worms (AW) after molting 4 times. After being mated, the pregnant female adults produce the newborn larvae (NBL) which pass into blood circulation and migrate to skeletal muscles, where they encapsulate to complete the life cycle [12]. Gut mucosal epithelium is the first native physical barrier to defense the IIL larval invasion, and it is also a principal interaction site between intestinal nematode and the host [13,14]. Gut mucosal immune response is crucial for developing anti-*Trichinella* vaccines to block larval invasion of gut mucosa, to interrupt IIL development to the AW stage and to expel residual IIL and AW from the gut [15,16]. Therefore, two novel *T*. *spiralis* genes (calreticulin and serine protease 1.1) in combination were used to construct oral DNA vaccines, and their induced protective immunity was evaluated in a murine model in the present study.

Calreticulin (CRT) is a highly conserved Ca^2+^-binding protein present in all organism cells except erythrocytes [17]. It has been found that calreticulin of *Schistosoma mansoni* metacercariae inhibited cell adhesion and phagocytosis by regulating intracellular and extracellular Ca^2+^ concentrations, thus participating in parasite immune evasion [18]. Calreticulin from human filarial *Brugia malayi* (BmCRT) participated in the establishment of filarial infection by suppressing C1q-mediated host immune response [19]. *T*. *spiralis* calreticulin (Ts-CRT) promoted the parasite immune escape and survival in host by directly binding to host’s complement C1q, suggesting that TsCRT is a potential candidate target for developing vaccine against trichinellosis [20]. However, there are no reports on the immune protective effects of Ts-CRT vaccination against *T*. *spiralis* infection in the literatures.

In this study, a novel *T*. *spiralis* calreticulin (TsCRT) (GenBank: KRY34215.1) was gained from *T*. *spiralis* draft genome [21]. TsCRT was expressed in various *T*. *spiralis* developmental stages (IIL, AW and ML) and mainly localized on the epicuticle of this nematode. A new *T*. *spiralis* serine protease 1.1 (TsSP1.1; GenBank: ACA28930.1) was identified by proteomics analysis of surface proteins of *T*. *spiralis* ML, TsSP1.1 was expressed in cuticle and excretion/secretion proteins of various *T*. *spiralis* stages [22]. In the process of *T*. *spiralis* infection, the surface proteins of the parasite are first exposed to host’s gut mucosa, and they might play a vital role in mediating larval intrusion and eliciting local gut mucosal immune response [23,24].

The aim of this study was to investigate gut local mucosal and systemic immune responses and protective efficacy elicited by oral vaccination with TsCRT+TsSP1.1 in BALB/c mice.

## Materials and methods

### Ethics statement

This work was conducted according to the National Guidelines for Experimental Animal Welfare (Minister of Science and Technology, the People’s Republic of China, 2006). All animal experiment procedures were authorized by the Life Science Ethics Committee, Zhengzhou University (No. SCXK 2020–0004).

### Parasite, bacteria and mice

*Trichinella spiralis* strain (ISS534) was collected from a naturally infected domestic pig in central China and passaged in mice in our laboratory. The attenuated *Salmonella typhimurium* ΔcyaSL1344 strain in which the cya gene has been deleted was gifted by the Key Laboratory of Animal Disease and Public Health, Henan University of Science and Technology. The bacteria were used as a carrier of the eukaryotic expression vectors harboring the fusion gene of TsCRT+TsSP1.1 [15]. BALB/c mice (female, 4 weeks old) were purchased from the Experimental Animal Center of Zhengzhou University (Zhengzhou, China), and fed in individual ventilated cage (IVC, Suzhou, China).

### Collection of diverse *T*. *spiralis* lifecycle stages

*T*. *spiralis* ML from infected murine skeletal muscles at 42 days post-infection (dpi) were recovered by artificial digestion method [25], intestinal infective larvae (IIL) and AW were isolated from infected mouse intestine at 6 hpi, 3 and 6 dpi, respectively [26,27]. The female adults at 6 dpi were cultivated in RPMI-1640 supplemented with 10% fetal bovine serum (FBS; Gibco) at 37°C in 5% CO_2_ for 24 h, and the NBL were recovered as previously reported [28].

### Preparation of rTsCRT/rTsSP1.1 and anti-rTsCRT and anti-rTsSP1.1 serum

Full-length TsCRT sequence (GenBank: KRY34215.1) was cloned, and recombinant pQE80L/TsCRT was transformed into *Escherichia coli* BL21 (Novagen, USA) [29,30]. A novel *T*. *spiralis* serine protease 1.1 gene (TsSP1.1; GenBank: ACA28930.1) was identified by proteomics analysis of *T*. *spiralis* ML surface proteins [22]. A function domain of TsSP1.1 cDNA sequence was also cloned and the pQE80L/TsSP1.1 was transformed into *E*. *coli* BL21 (Novagen). Expression of rTsCRT and rTsSP1.1 was induced with 1 mM IPTG at 25°C for 8 h [31,32]. A Ni-NTA-Sefinose resin containing His tag (Sangon Biotech, Shanghai, China) was used to purify rTsCRT and rTsSP1.1 [33,34].

Two groups of female mice (15 mice per group) were subcutaneously immunized using 20 μg rTsCRT emulsified with ISA201 (Seppic, France) or 20 μg rTsSP1.1 with complete Freund’s adjuvant, respectively. Two booster immunizations were performed with 20 μg rTsCRT emulsified with ISA201 or 20 μg rTsSP1.1 with incomplete Freund’s adjuvant at a 14-day interval [35,36]. At two weeks following the third immunization, tail blood was taken and immune sera against rTsCRT or rTsSP1.1 were isolated and stored at– 80°C till use.

### Construction of recombinant expression plasmids

Full-length TsCRT gene (GenBank: KRY34215.1) was acquired by PCR amplification with the following primers carrying BamHI and EcoRI **(bold**) (5′-CGC**GGATCC**GCCA CCATGGAGGTTTATTTGAAAGAAACGTTCG-3′, 5′-CCG**GAATTC**TTAAAGTTCGTC GTCAGCATGTTTC-3′). The function domain of TsSP1.1 gene (GenBank: ACA28930.1) was obtained by PCR amplification with the following primers containing HindIII and EcoRI **(bold**) (5′-C**AAGCTT**GCCACCATGATCGTTGGTGGATGGGTTGCAAAG-3′, 5′-CCG **GAATTC**CTATTGGTTGTATATCCATTTTAC-3′), and the fusion gene (TsCRT+TsSP1.1) was synthesized by Dongxuan Gene Technology Co., Ltd (Kunshan, China). The amplified DNA fragments were respectively cloned into the pcDNA3.1 (Invitrogen, Carlsbad, USA). The recombinant pcDNA3.1-TsCRT+TsSP1.1, pcDNA3.1-TsCRT, pcDNA3.1-TsSP1.1 and empty control plasmid pcDNA3.1 were electroporated into the attenuated *S*. *typhimurium* ΔcyaSL1344 strain in a 2-mm cuvette (Gene Pulser Xcell, Bio-Rad, CA, USA) as reported before [37]. The positive transformant was selected on MacConkey Agar Medium with 50 μg/ml ampicillin and identified by PCR. The PCR products were sequenced (Sangon Biotech, Shanghai, China) to further verify the true introduction of plasmids into the bacteria and the successful construction of oral DNA vaccines [9].

### Detection of the *in vitro* expression of recombinant plasmids by RT-PCR and indirect immunofluorescence test (IIFT)

Human embryonic kidney cells (293T) were cultivated in DMEM medium containing 100 U/ml penicillin, 100 μg/ml streptomycin and 10% fetal bovine serum (FBS; Gibco, New Zealand) at 37°C in 5% CO_2_. When the cells were grown to 90% confluence, the cells were transfected with pcDNA3.1-TsCRT+TsSP1.1, pcDNA3.1-TsCRT, pcDNA3.1-TsSP1.1, pcDNA3.1 using a Lipofectamine 2000 (Invitrogen, USA) at 37°C for 48 h. Total RNAs were extracted from transfected cells, and mRNA transcription level was ascertained by RT-PCR with the above-mentioned specific primers as previously described [38]. The protein expression in TsCRT+TsSP1.1, TsCRT, TsSP1.1 DNA-transfected 293T cells was investigated using IIFT as reported before [37]. Briefly, 293T cells were fixed with cold acetone at room temperature for 20 min. Following being washed with PBS, the cells were permeabilized using 0.1% TritonX-100 for 15 min, blocked using 5% normal goat serum at 37°C for 1 h, and followed by the incubation of anti-rTsCRT serum and anti-rTsSP1.1 serum (1:10) at 4°C overnight. After washes again, the cells were dyed using FITC-anti-mouse IgG conjugate (1:100; Santa Cruz, USA) at 37°C for 1 h. The cells were re-dyed using 4′, 6-diamidino-2-phenylindole (DAPI; Sangon Biotech, Shanghai, China) for 7 min, and examined under fluorescence microscopy (Olympus, Japan) [39]

### Western blotting of the *in vitro* expression of recombinant plasmids

Soluble proteins of transfected 293T cells were analyzed and identified on Western blotting analysis as previously reported [40,41]. In brief, the proteins were separated on SDS-PAGE and transferred onto nitrocellulose (NC) membrane (Millipore, USA) in a semi-dry transfer cell (Bio-Rad, USA) [42,43]. The membrane was blocked with 5% skim milk at 37°C for 2 h, and cut into strips. The strips were probed using various sera (1:100; anti- rTsCRT serum, anti-rTsSP1.1 serum and normal serum) at 37°C for 2 h. After washing with TBST, the strips were incubated with HRP-conjugated anti-mouse IgG (1:10000; Southern Biotech) at 37°C for 1 h. After washing again, the strips were developed with 3, 3’-diaminobenzidine tetrahydrochloride (DAB; Sigma-Aldrich, USA) and stopped by washing the membrane with deionized water [44]

### Vaccination schedules of oral DNA vaccine and sample collection

**Fig 1:**
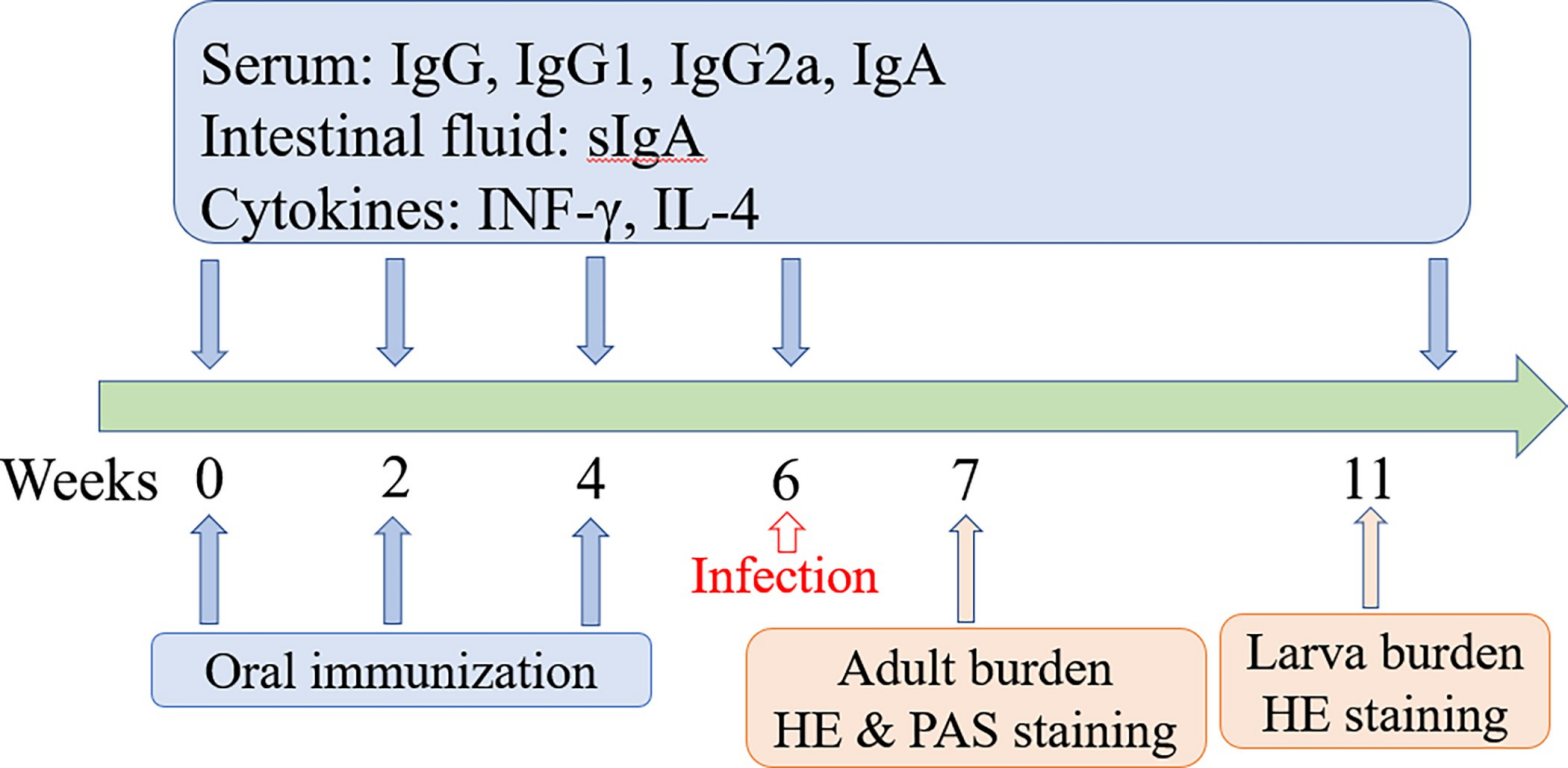
The vaccination scheme and detection protocol designed in this study. Oral vaccination of mice with DNA vaccine was administered three times (weeks 0, 2 and 4). Five mice of each group were euthanized at week 0, 2, 4 and 6 after vaccination, levels of intestinal sIgA and cytokines (IFN-γ and IL-4) were determined by ELISA. The vaccinated mice were orally challenged with 300 *T*. *spiralis* ML two weeks following the final vaccination. At weeks 7 and 11 after vaccination (e.g., 7 and 35 days after challenge), ten mice of each group were sacrificed and intestinal adult worm, female fecundity and muscle larval burden (larvae per gram, LPG) were assessed to evaluate the protective efficacy induced by vaccination with DNA vaccine. Pathological changes of intestines and muscles from infected mice were examined under microscopy at 7 and 35 days after challenge.

Two hundred mice were randomly divided into five groups (40 mice per group). Pre-immune sera were collected through tail bleeding before vaccination. Each mouse of vaccine group was inoculated orally with 1 × 10^8^ CFU recombinant bacteria of pcDNA3.1-TsCRT+TsSP1.1, pcDNA3.1-TsCRT or pcDNA3.1-TsSP1.1, respectively. Two control groups were administrated with ΔcyaSL1344/pcDNA3.1 alone or only PBS. The PBS received group could be considered as the infection control group. The vaccination was boosted two times at a 2-week interval. At 30 min prior to oral vaccination, 100 μl of 10% NaHCO_3_ were orally administrated for all mice to neutralize the gastric acids. At weeks 0, 2, 4 and 6 post-vaccination, five mice of each group were euthanatized; serum, spleen, mesenteric lymph nodes (MLN), Peyer’s patches (PP), and intestinal washing fluid were collected to investigate the levels of immune response to oral DNA vaccination [45,46]. To assess the adult burden and female reproductive capacity (the *in vitro* production of NBL deposited by each female for 72 h), the adult worms were recovered from intestine of all infected mice one week after challenge (e.g., 7 weeks after vaccination). The vaccination protocol scheme of this study was shown in Fig 1.

### RT-PCR and IIFT for the *in vivo* expression of recombinant plasmid

Transcription and expression of TsCRT+TsSP1.1, TsCRT and TsSP1.1 in vaccinated murine spleens and MLN were investigated by RT-PCR two weeks after the first vaccination. RNAs of spleens and MLN were isolated using Trizol regents (Invitrogen, Carlsbad, USA). The PCR products were analyzed on 1% agarose gels, murine β-actin was also amplified as the housekeeping gene control [47].

To evaluate the protein expression of TsCRT+TsSP1.1, TsCRT and TsSP1.1, 2 μm-thick cross-sections of spleen and MLN were cut by a microtome. The sections were blocked with 5% normal goat serum at 37°C for 1 h. After washing with PBS, the sections were incubated at 4°C overnight using anti-rTsCRT serum, anti-rTsSP1.1 serum and normal serum. Following washing again, the sections were probed with FITC-conjugated anti-mouse IgG (1:100; Santa Cruz), and observed under a fluorescence microscope (Olympus, Japan) [48,49].

### Challenge infection experiments

To evaluate the immune protection produced by immunization with TsCRT+TsSP1.1, TsCRT and TsSP1.1, each vaccinated mouse was orally challenged with 300 *T*. *spiralis* ML two weeks following the last vaccination. The AWs were collected from the gut of ten vaccinated mice from each group at 7 dpi [50]. The ML was obtained from remaining ten mice from each group at 35 dpi by artificial digestion of infected murine skeletal muscles [25,51]. The immune protective effect induced by TsCRT+TsSP1.1, TsCRT and TsSP1.1 immunization was ascertained as the worm burden reduction of intestinal AWs and muscle larvae per gram (LPG) of muscle tissues from immunized mice compared to the PBS group [14,52]. Additionally, the female fecundity was also ascertained in immunized mice and control groups [53].

### ELISA determination of serum specific anti-*Trichinella* antibodies

Serum specific antibody responses (total IgG, IgG1, IgG2a and IgA) of all vaccinated mice were determined by ELISA which is a modification of the previously described [54–56]. Briefly, the ELISA plate was coated at 4°C overnight with 2 μg/ml of ML soluble crude antigen. The plate was blocked using 5% skimmed milk at 37°C for 1 h. After being washed with PBST, the plate was probed at 37°C for 1 h with diverse diluted immune sera (1:100 for detecting IgG, 1:50 for detecting IgG1, IgG2a and IgA). Following washes again, the plate was incubated at 37°C for 1 h with HRP-conjugated anti-mouse IgG, IgG1/IgG2a and IgA (1:10000; Southern Biotech, USA), then colored using the substrate OPD (Sigma-Aldrich) plus 0.15% H_2_O_2_, and reaction was stopped by using 2 M H_2_SO_4_. The absorbance (OD value) at 492 nm was determined using a microplate reader (Tecan, Schweiz, Switzerland). The cut-off value of the ELISA was evaluated based on a 2.1-fold increase over the average OD value of the negative serum samples from normal unimmunized mice. Ratios *<*2.1 of the samples to be tested/negative sample (OD values of the samples to be tested divided by OD of the negative, S/N *<* 2.1) were regarded as negative, whereas S/N ≥2.1 was regarded as positive.

### Assay of intestinal sIgA and histamine

To assess total and *Trichinella*-specific sIgA in gut fluid, gut washing fluid was collected as previously described [57,58]. Briefly, a 20 cm long of intestinal segment was obtained, and the gut interior was washed 3 times with 1 ml of cold PBS with 1% protease inhibitor cocktail (Sangon Biotech, Shanghai, China). The washing fluid was recovered, and total enteral sIgA was measured with a sandwich ELISA as previously reported [37]. *Trichinella*-specific sIgA was assayed by ELISA using 2 μg/ml of ML soluble crude antigen. Coloration was developed using OPD and the OD value at 492 nm was determined as previously reported [59].

As the histamine secreted by intestinal mucosal mast cells plays a prominent role in intestinal inflammation and adult worm expulsion, histamine levels in gut fluids were assessed at weeks 0, 2, 4 and 6 after vaccination, and at 5 weeks following larval challenge. The levels of intestinal histamine concentrations were measured using a mouse enzyme-linked immunosorbent assay (ELISA) kit according to the manufacturer’s instructions (Elabscience, Biotechnol, Wuhan, China). The results were shown in ng/ml ± SD. All samples were in duplicate [60,61].

### ELISA determination of cytokine responses

To evaluate *Trichinella*-specific cellular immune responses, five mice of each group were euthanized at week 0, 2, 4, 6 after vaccination and 5 weeks following challenge. The spleens, MLN and PP were recovered from all vaccinated mice, homogenized in complete DMEM medium (Gibco, Auckland, New Zealand). The pellets were obtained after centrifugation at 300 g for 5 min, and the cells were isolated as reported [62,63]. The cell density was adjusted to 2 × 10^6^ cells/ml in DMEM medium with 5% FBS, penicillin (100 U/ml) and streptomycin (100 μg/ml). These cells were stimulated by 2 μg/ml of ML soluble crude antigen for 72 h at 37°C and 5% CO_2_ [64,65], The supernatant was collected and two cytokines (IFN-γ and IL-4) were assayed by a sandwich ELISA kit (BD Biosciences Pharmingen, USA). Cytokine concentration was presented as picograms per milliliter (pg/ml).

### Antibody-dependent cell-mediated cytotoxicity (ADCC) assay

Specific antibody mediated cytotoxicity on the NBL was performed as previously reported [66]. Briefly, 100 NBL were cultured with 2 × 10^5^ murine peritoneal exudate cells (PECs) in a 96-well plate with DMEM medium supplemented with various kinds of immune serum (1:50–1:800 dilutions) at 37°C for 72 h, *Trichinella-*infected mouse serum was used as positive control, mouse serum from the pcDNA3.1 and PBS control groups as negative controls. After being cultured for 72 h, the larval viability was assessed according to their morphology and activity. The living NBL was active and mobile, while the dead NBL was inactive and straight. Cytotoxicity was defined as the percentage of dead NBL to the total larvae observed in each assay [43,67].

### Small intestine and muscle pathological examination

To evaluate the pathological change of intestine and muscles, small intestine and masseter muscles were collected from infected mice at 7 and 35 dpi, and fixed in 4% formalin for 24 h and embedded in paraffin wax, 3-μm-thick muscle sections were prepared, deparaffinized and stained using hematoxylin and eosin (HE) stain and periodic acid-schiff stain (PAS) [45]. Gut mucosa of different groups of infected mice were examined under light microscopy, and enteral villus width and the numbers of intestinal goblet cells per field (400×) were examined and numbered. The encapsulated larvae per field (100 ×) and inflammatory cells (eosinophils, neutrophils and lymphocytes) per field (400 ×) on the muscle sections were numbered as previously described [68].

### qPCR assay of mucin 2 mRNA expression

Total RNAs from small intestinal tissues of infected mice were isolated using Trizol reagent (Invitrogen, USA). Contamination with genomic DNA samples was avoided by treatment with DNase (Thermo Fisher, USA). Mucin 2 (Muc2) mRNA expression level was assessed using qPCR as described previously [69]. The primers of mucin 2 were as follow: 5′-TGTGGCCTGTGTGGGAACTTT-3′ and 5′-GGCCCGAGAGTAGACCTTGG-3′ [70]. Relative level of mucin 2 mRNA expression was normalized by subtracting the mRNA expression level of a murine housekeeping gene glyceraldehyde-3-phosphate dehydrogenase (GAPDH, GenBank: NM_001289726.1), and then calculated in line with comparative Ct (^2−ΔΔCt^) method [10,71]. Each experiment had three replicates.

### Statistical analysis

All the data were statistically analyzed with SPSS for Windows, version 21.0. The data were shown as the mean ± standard deviation (SD). Differences among various groups were analyzed by a One-way ANOVA and Student’s t test. The correlation analysis was used to ascertain the relationship between ADCC cytotoxicity and antibody dilution/culture time. *P* < 0.05 was defined as statistical significance.

## Results

### Identification of recombinant plasmids

**Fig 2:**
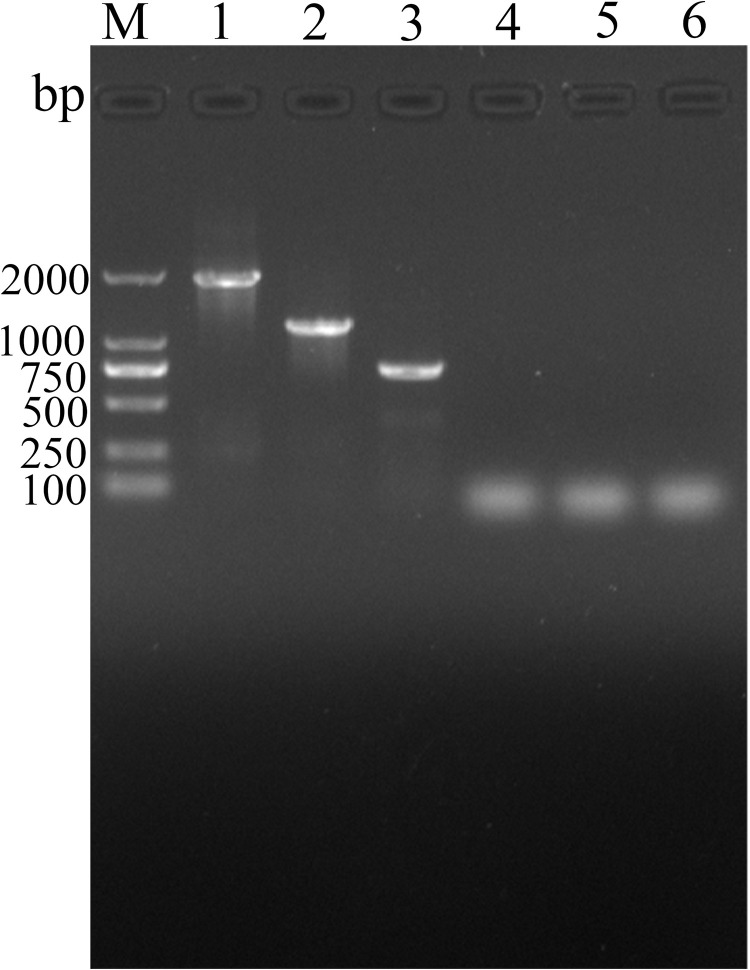
PCR identification of recombinant plasmids. Lane M: DNA marker. Lane 1: PCR product of pcDNA3.1-TsCRT+TsSP1.1. Lane 2: PCR product of pcDNA3.1-TsCRT. Lane 3: PCR product of pcDNA3.1-TsSP1.1. Lane 4: PCR product of pcDNA3.1 using TsCRT+TsSP1.1 primers. Lane 5: PCR product of pcDNA3.1 with TsCRT primers. Lane 6: PCR product of pcDNA3.1 with TsSP1.1 primers.

Electrophoresis of PCR products showed that three recombinant plasmids contained three inserts of about 1908, 1179 and 745 bp, respectively (Fig 2). Sequence analysis indicated that the amplified fragments of TsCRT+TsSP1.1, TsCRT and TsSP1.1 gene consisted of 1908, 1179 and 745 bp, respectively; TsCRT and TsSP1.1 had a 100 and 99.58% identity to those of TsCRT (KRY34215.1) and TsSP1.1 (ACA28930.1) in GenBank, respectively.

### The *in vitro* expression of recombinant plasmids

**Fig 3:**
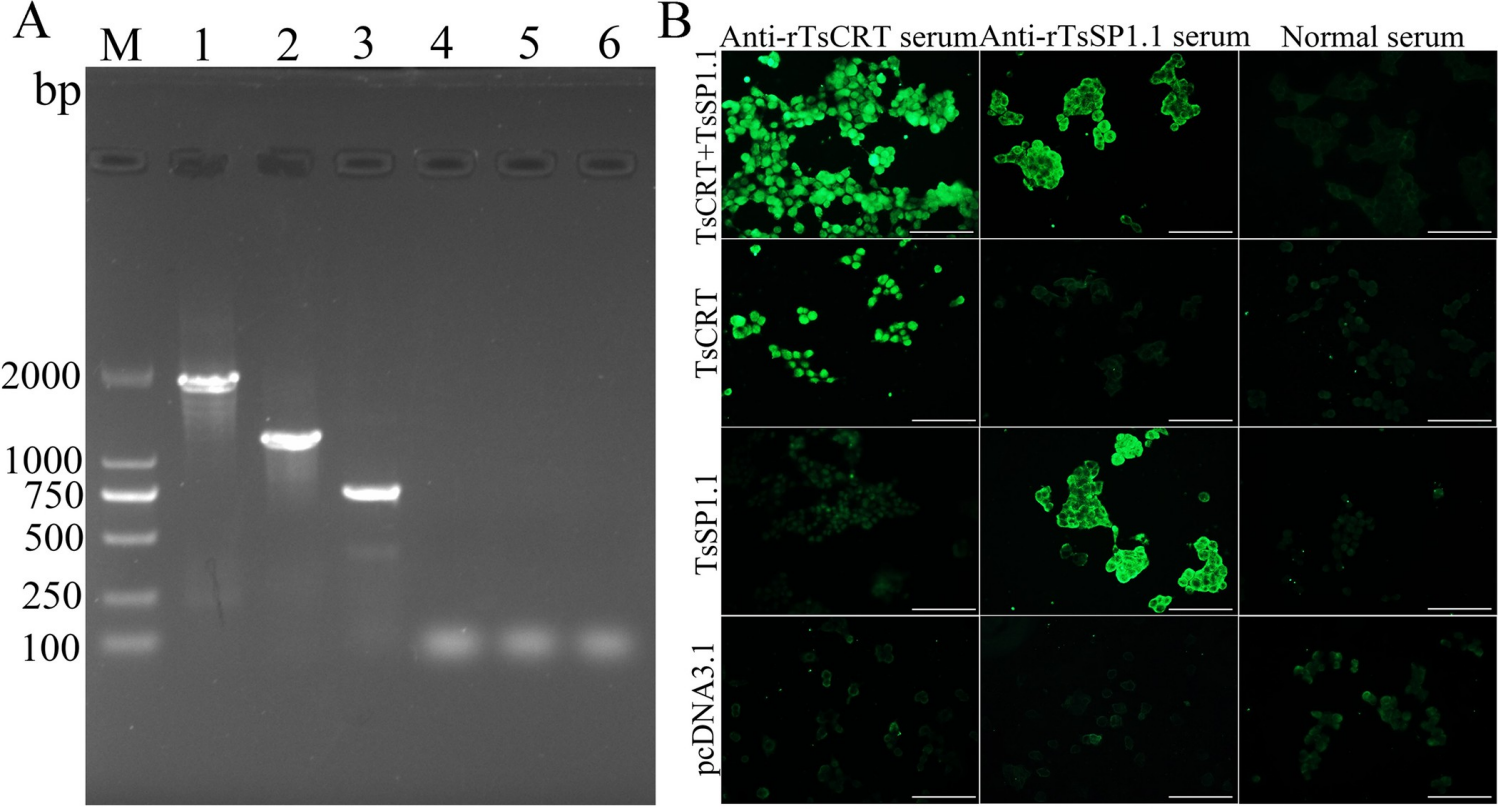
The *in vitro* transcription and expression of TsCRT+TsSP1.1, TsCRT and TsSP1.1. **A:** Transcription of TsCRT+TsSP1.1, TsCRT and TsSP1.1 in 293T cells were analyzed by RT-PCR. Lane M: DNA marker. Lane 1: pcDNA3.1-TsCRT+TsSP1.1 transfected cells. Lane 2: pcDNA3.1-TsCRT transfected cells. Lane 3: pcDNA3.1-TsSP1.1 transfected cell. Lane 4: only pcDNA3.1 transfected cells amplified by TsCRT+TsSP1.1 primers. Lane 5: pcDNA3.1 transfected cells amplified by TsCRT primers. Lane 6: pcDNA3.1 transfected cells amplified by TsSP1.1 primers. **B:** Protein expression of TsCRT+TsSP1.1, TsCRT and TsSP1.1 in transfected cells was detected by IIFT using with anti-rTsCRT serum and anti-rTsSP1.1 serum, but not in only pcDNA3.1- transfected cells. Scale bar: 200 μm.

The *in vitro* transcription of TsCRT+TsSP1.1, TsCRT and TsSP1.1 gene in 293T cells was analyzed by RT-PCR. The results revealed that three amplified DNA fragments were observed in transfected cells, respectively, but not in only pcDNA3.1-transfected cells (Fig 3A). The protein expression of TsCRT+TsSP1.1, TsCRT and TsSP1.1 in transfected cells was detected by IIFT using anti-rTsCRT serum and anti-rTsSP1.1 serum, but not in empty pcDNA3.1 transfected cells (Fig 3B).

**Fig 4:**
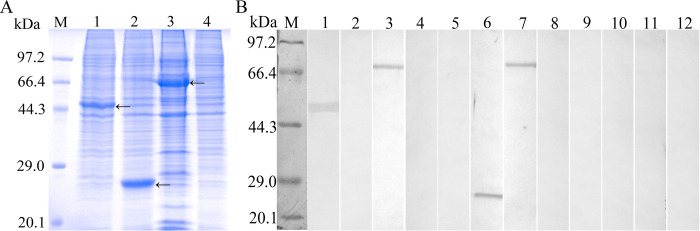
Western blotting of the *in vitro* expression of TsCRT+TsSP1.1, TsCRT and TsSP1.1. **A:** SDS-PAGE analysis of soluble proteins of transfected 293T cells. Lane M: protein marker. Lane 1: pcDNA3.1-TsCRT transfected cells. Lane 2: pcDNA3.1-TsSP1.1 transfected cells. Lane 3: pcDNA3.1-TsCRT+TsSP1.1 transfected cells. Lane 4: empty pcDNA3.1 transfected cells. **B**: Western blot analysis of protein expression of TsCRT (lane 1, 5, 9), TsSP1.1 (lane 2, 6, 10) and TsCRT+TsSP1.1 (lane 3, 7, 11) in transfected cells by anti-rTsCRT serum (Lane 1–4), anti-rTsSP1.1 serum (Lane 5–8) and normal serum (Lane 9–12). No protein bands of pcDNA3.1 transfected cells (lane 4, 8, and 12) were identified by anti-rTsCRT serum (Lane 4), anti-rTsSP1.1 serum (Lane 8) and normal serum (Lane 12). Three expressed protein bands of TsCRT, TsSP1.1 and TsCRT+TsSP1.1 with about 47, 26.07 and 71.53 kDa were indicated by arrows.

Furthermore, the results of Western blotting analysis revealed that three individual protein bands of recombinant TsCRT+TsSP1.1, TsCRT and TsSP1.1 with about 71.53, 47 and 26.07 kDa were recognized by anti- rTsCRT serum, anti-rTsSP1.1 serum and infection serum, but no bands were recognized in soluble proteins of only pcDNA3.1-transfected cells (Fig 4).

### The *in vivo* expression of recombinant plasmids

**Fig 5:**
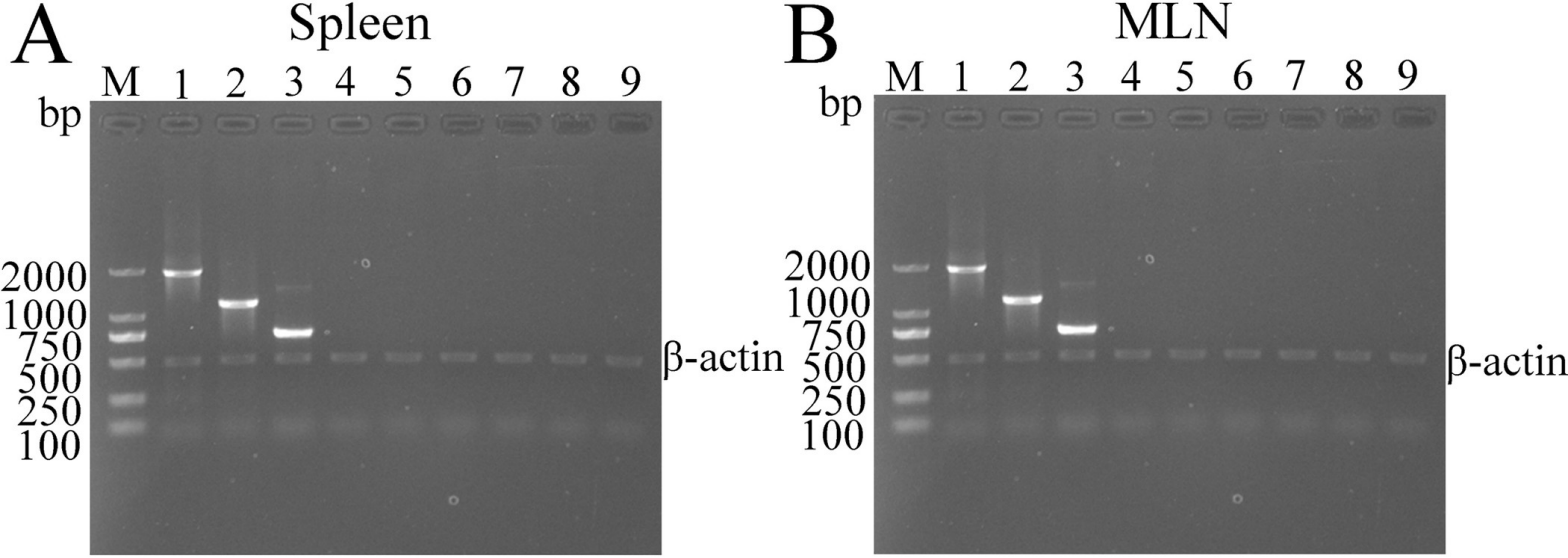
The *in vivo* transcription of TsCRT+TsSP1.1, TsCRT and TsSP1.1 in spleen (A) and MLN (B) of immunized mice were analyzed by RT-PCR. Lane M: DNA markers; Lane 1: TsCRT+TsSP1.1 immunized murine RNAs amplified by TsCRT+TsSP1.1 primers. Lane 2: TsCRT immunized murine RNAs amplified by TsCRT primer. Lane 3: TsSP1.1 immunized murine RNAs amplified by TsSP1.1 primer. Lane 4: pcDNA3.1 immunized murine RNAs amplified by TsCRT+TsSP1.1 primers. Lane 5: pcDNA3.1 immunized murine RNAs by TsCRT primer. Lane 6: pcDNA3.1 immunized murine RNAs by TsSP1.1 primer. Lane 7: PBS inoculated murine RNAs amplified by TsCRT+TsSP1.1 primers. Lane 8: PBS inoculated murine RNAs by TsCRT primer. Lane 9: PBS inoculated murine RNAs by TsSP1.1 primer.

**Fig 6:**
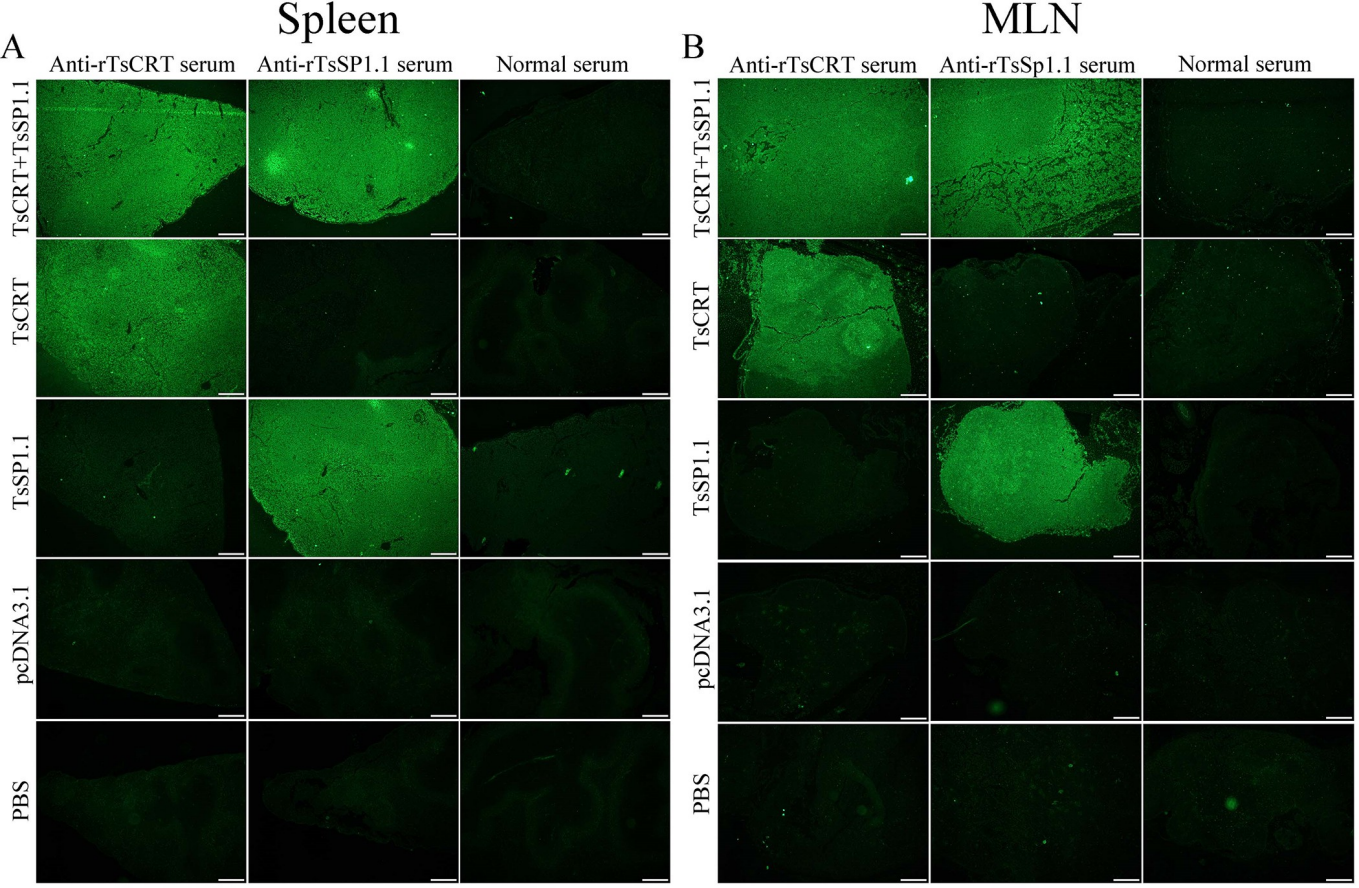
The *in vivo* expression of TsCRT+TsSP1.1, TsCRT and TsSP1.1 in spleen and MLN of immunized mice were assessed using IIFT. IIFT with anti-rTsCRT and anti-rTsSP1.1 sera showed that immunostaining was observes in vaccinated murine spleen (**A**) and MLN (**B)** sections, but not in spleen and MLN sections probed using normal serum. No immunostaining in spleen and MLN sections of mice inoculated with only empty pcDNA3.1 and PBS alone was detected by immune serum. Scale bar: 200 μm.

Total RNAs were isolated from vaccinated murine spleen and MLN at 2 weeks after the first vaccination, transcription of TsCRT+TsSP1.1, TsCRT and TsSP1.1 in murine tissues was ascertained by RT-PCR. The results revealed that TsCRT+TsSP1.1, TsCRT and TsSP1.1 gene was transcribed in spleen and MLN from immunized mice, but not in those from mice inoculated with only pcDNA3.1 or PBS (Fig 5). The IIFT showed that the immunostaining was detected in spleen and MLN sections of immunized mice with TsCRT+TsSP1.1, TsCRT and TsSP1.1, but not in those from the pcDNA3.1 and PBS group (Fig 6). Furthermore, when the spleen and MLN sections from immunized mice were probed with normal serum, no immunostaining was observed.

### Serum anti-*Trichinella* antibody responses in immunized mice

The mice were orally vaccinated three times with TsCRT+TsSP1.1, TsCRT or TsSP1.1, serum anti-*Trichinella* antibody IgG titers two weeks after the final vaccination were measured by ELISA using ML soluble crude antigen. Anti-*Trichinella* IgG levels in all the vaccinated mice were significantly increased, and mean antibody titer of three immunized groups achieved to 1: 10^5^ after the final vaccination, respectively (S1 Fig), indicated that TsCRT+TsSP1.1, TsCRT and TsSP1.1 had a good immunogenicity. However, anti- *Trichinella* IgG antibody responses were not detected in mice vaccinated with only pcDNA3.1 and PBS.

**Fig 7:**
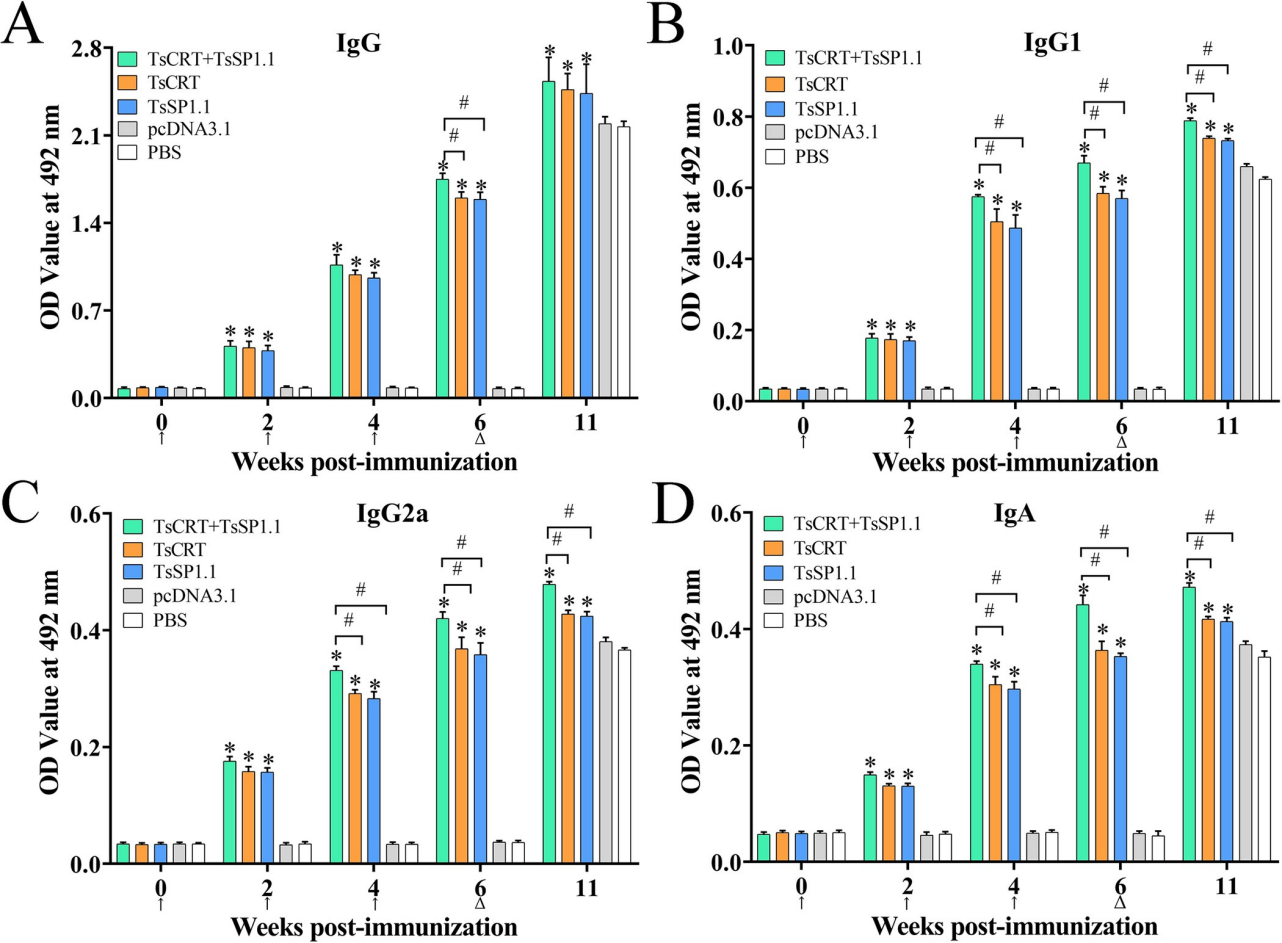
Serum anti-*Trichinella* antibody responses in mice vaccinated with TsCRT+TsSP1.1, TsCRT and TsSP1. **1**. Specific total anti-*Trichinella* IgG response was assessed in mice vaccinated with TsCRT+TsSP1.1, TsCRT or TsSP1.1 at various times following vaccination (**A**). Specific IgG1 (**B**) and IgG2a (**C**) subclass responses were also ascertained at various times after vaccination. **D:** IgA levels in vaccinated mice. The OD values from each group are presented as mean ± SD of antibody levels (n = 10). The vaccination times are shown with arrows (↑) and the challenge time is indicated by triangles (△). *P < 0.05 compared to the PBS group. ^#^*P* < 0.05 compared among various immunization groups.

Anti-*Trichinella* IgG levels of three immunized groups were significantly higher than that of pcDNA3.1 and PBS control groups at 2, 4 and 6 weeks after vaccination (*F*_2W_ = 240.62, *F*_4W_ = 1278.35, *F*_6W_ = 4789.42, *P* < 0.0001). However, anti-*Trichinella* IgG levels in two control groups also increased at 5 weeks after larval challenge (Fig 7A). Both IgG1 and IgG2a levels of three immunized groups were also obviously higher than two control groups at 2, 4 and 6 weeks following vaccination and 5 weeks after challenge (*P* < 0.05) (Fig 7B and 7C). Moreover, the IgG1 level of three immunized groups at 2, 4 and 6 weeks after vaccination was obviously higher than IgG2a level (*t*_2W_ = 3.070, *t*_4W_ = 22.574, *t*_6W_ = 20.969; *P* < 0.01), indicating that oral vaccination with TsCRT+TsSP1.1, TsCRT and TsSP1.1 induced a mixed Th1/Th2 immune response with Th2 predominance. Furthermore, anti-*Trichinella* antibody IgA was also measured, the results showed that IgA levels were significantly elevated in three immunized groups compared to the pcDNA3.1 and PBS control groups (*F*_2w_ = 1138.89, *F*_4W_ = 2649.79, *F*_6W_ = 2990.70, *P <* 0.0001) (Fig 7D). Additionally, specific IgG (IgG1/IgG2a) and IgA levels of TsCRT+TsSP1.1 immunized group at 6 weeks after vaccination were evidently higher than immunized group with only TsCRT or TsSP1.1 alone (*F*_IgG_ = 31.544, *F*_IgG1_ = 71.117, *F*_IgG2a_ = 36.491, *F*_IgA_ = 137.350, *P* < 0.05). The results demonstrated that combined vaccination of mice with TsCRT+TsSP1.1 triggered a stronger IgG and IgA antibody responses than either of individual TsCRT or TsSP1.1.

Moreover, anti-*Trichinella* IgG levels of three immunized groups (TsCRT+TsSP1.1, TsCRT and TsSP1.1) had statistically significant differences at 4, 6 and 11 weeks after vaccination (*F*_TsCRT+TsSP1.1_ = 352.404, *F*_TsCRT_ = 827.685, *F*_TsSP1.1_ = 277.654, *P* < 0.001). Furthermore, Both IgG1 and IgG2a levels of three immunized groups also had statistical differences at 4, 6 and 11 weeks after vaccination (IgG1: *F*_TsCRT+TsSP1.1_ = 712.661, *F*_TsCRT_ = 260.677, *F*_TsSP1.1_ = 247.484, *P* < 0.001; IgG2a: *F*_TsCRT+TsSP1.1_ = 832.303, *F*_TsCRT_ = 297.189, *F*_TsSP1.1_ = 252.951, *P* < 0.001). Additionally, there were statistically significant differences of IgA antibody levels of three immunized groups at 4, 6 and 11 weeks after vaccination (*F*_TsCRT+TsSP1.1_ = 448.398, *F*_TsCRT_ = 212.459, *F*_TsSP1.1_ = 448.146, *P* < 0.001). The results demonstrated that specific IgG (IgG1/IgG2a) and IgA levels in three immunized groups gradually elevated after vaccination, and further increased after challenge infection.

### Intestinal mucosal immune response

**Fig 8:**
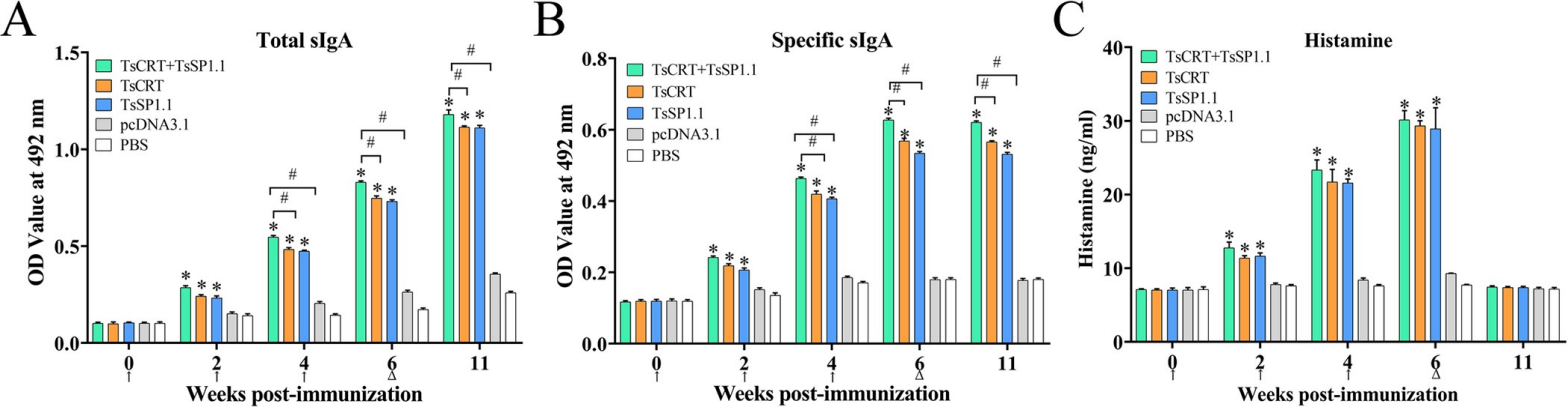
**Levels of total sIgA (A), *Trichinella*-specific sIgA (B) and histamine (C) in gut washes of vaccinated mice.** The data are shown as the mean OD values ± SD for five mice per group. No notable specific sIgA response was detected in the pcDNA3.1 and PBS control groups. The vaccination times are shown with arrows (↑) and the challenge time is indicated by triangles (△). *P < 0.0001 compared with PBS group. ^#^*P* < 0.001 compared among various immunization groups.

At 6 weeks following vaccination, total sIgA level in gut fluid of mice immunized with TsCRT+TsSP1.1, TsCRT and TsSP1.1 was significantly higher than those of mice inoculated with only pcDNA3.1 or PBS group (*F* = 1226.49, *P* < 0.0001) (Fig 8A). *Trichinella*-specific sIgA levels in three immunized groups were also distinctly higher than the pcDNA3.1 or PBS control group (*F* = 1601.83, *P* < 0.0001) (Fig 8B). The higher levels of total and specific sIgA in three immunized groups maintained to 5 weeks after challenge (*F*_total_ = 6061.89, *F*_specific_ = 1642.00, *P* < 0.0001). No specific mucosal sIgA responses were observed in mice inoculated with only pcDNA3.1 and PBS alone. Furthermore, specific sIgA levels of TsCRT+TsSP1.1 immunized group at 4, 6 and 11 weeks after vaccination were evidently higher than individual TsCRT or TsSP1.1 vaccination (*F*_4w_ = 214.566, *F*_6w_ = 654.441, *F*_11w_ = 550.582, *P* < 0.05), suggesting that mixed vaccination of mice with TsCRT+TsSP1.1 produced a stronger gut mucosal sIgA response than either of single TsCRT or TsSP1.1 DNA vaccination.

Histamine contents of gut washing fluid at various times after immunization and challenge were measured by ELISA kit. The results showed that compared to the PBS group, the histamine level of three groups of mice immunized with TsCRT+TsSP1.1, TsCRT and TsSP1.1 were significantly increased at 2, 4 and 6 weeks after the first immunization (*F*_2w_ = 141.546, *F*_4w_ = 297.197, *F*_6w_ = 325.944, *P* < 0.0001). However, intestinal histamine level at 5 weeks after challenge has evidently regressed to the normal level of control groups (Fig 8C). The results suggested that oral immunization of mice with TsCRT+TsSP1.1, TsCRT and TsSP1.1 elicited an obvious intestinal mucosal response and histamine secretion.

### Cytokine expression levels of immunized mice

**Fig 9:**
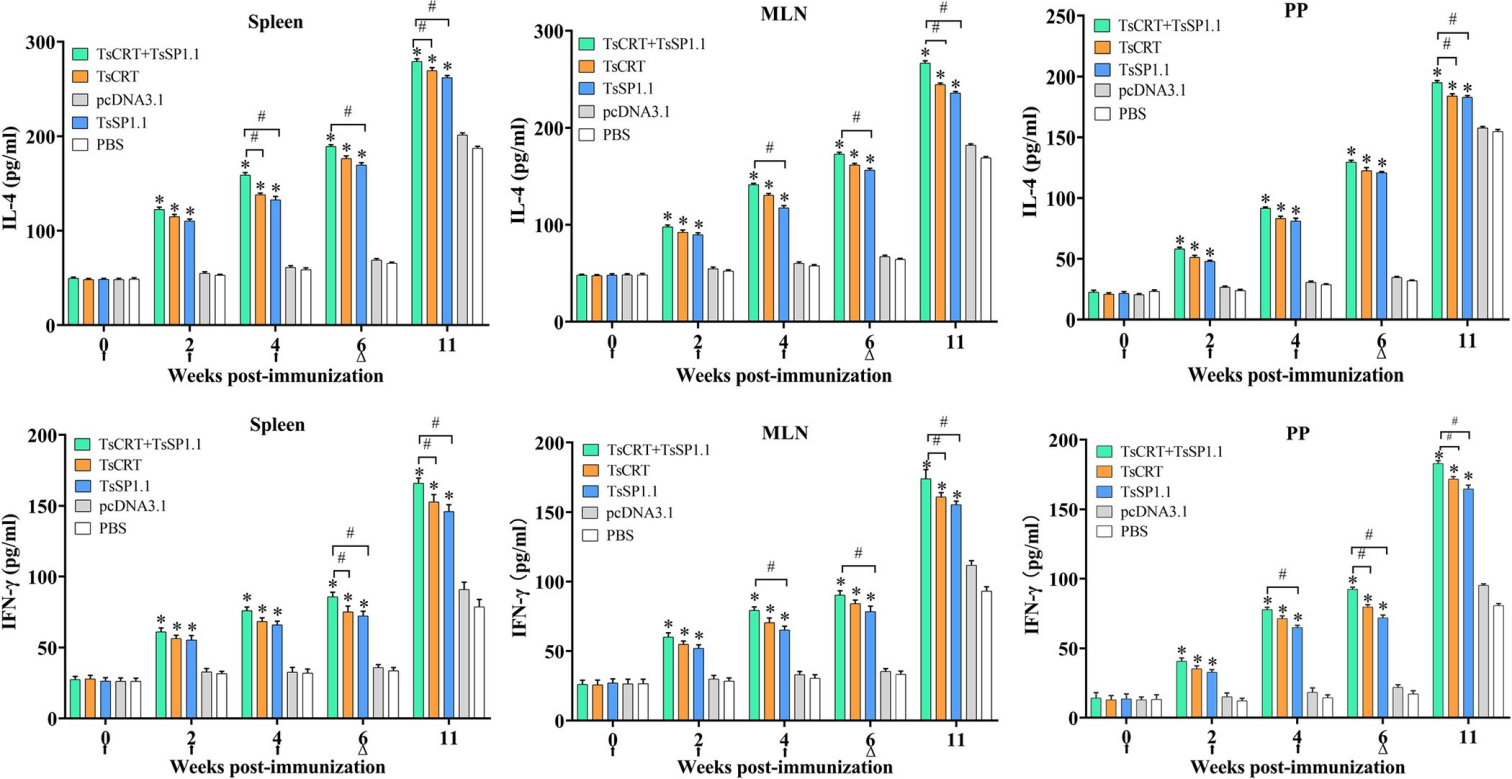
Cytokines secreted by spleen, mesenteric lymph nodes (MLN) and Peyer’s patches (PP) from mice immunized with TsCRT+TsSP1.1, TsCRT or TsSP1.1 at different times after immunization. Concentrations of two cytokines (IFN-γ and IL-4) were measured in supernatant after the spleen, MLN and PP cells were stimulated with 2 μg of ML soluble crude antigens for 72 h at 37°C and 5% CO_2_. The data are shown as the mean ± SD of five mice per group. The vaccination times are shown with arrows (↑) and the challenge time is indicated by triangles (Δ). * *P* < 0.001 compared to the PBS control groups. ^#^*P* < 0.05 compared among various vaccination groups.

The ELISA results showed that the cytokine levels of Th1 (IFN-γ) and Th2 (IL-4) in five groups of mice had no significant difference before vaccination (*P* > 0.05). But at 2, 4 and 6 weeks after vaccination, the levels of two cytokines in three groups of mice immunized with TsCRT+TsSP1.1, TsCRT or TsSP1.1 were remarkably increased compared to the PBS group (2w: *F*_IFN-γ_ = 164.973, *F*_IL-4_ = 1520.131, *P* < 0.001; 4w: *F*_IFN-γ_ = 281.999, *F*_IL-4_ = 1892.823, *P* < 0.001; 6w: *F*_IFN-γ_ = 310.873, *F*_IL-4_ = 4658.229, *P* < 0.001). Moreover, the levels of IFN-γ and IL-4 in three immunized groups were further elevated at five weeks after challenge (11 weeks following vaccination) (*F*_IFN-γ_ = 336.391, *F*_IL-4_ = 1378.752, *P* < 0.001) (Fig 9). Additionally, the levels of IFN-γ and IL-4 of TsCRT+TsSP1.1 immunized group at 6 after vaccination and 5 weeks following challenge were notably higher than individual TsCRT or TsSP1.1 vaccination (*P* < 0.05). The results indicated that vaccination with TsCRT+TsSP1.1, TsCRT and TsSP1.1 triggered the concomitant Th1/Th2 responses, and suggested that oral immunization of mice with TsCRT+TsSP1.1, TsCRT and TsSP1.1 evoked both systemic (spleen) and intestinal mucosal local (MLN and PP) cellular immune responses.

### Immune protection of vaccination with TsCRT+TsSP1.1, TsCRT and TsSP1.1

**Fig 10:**
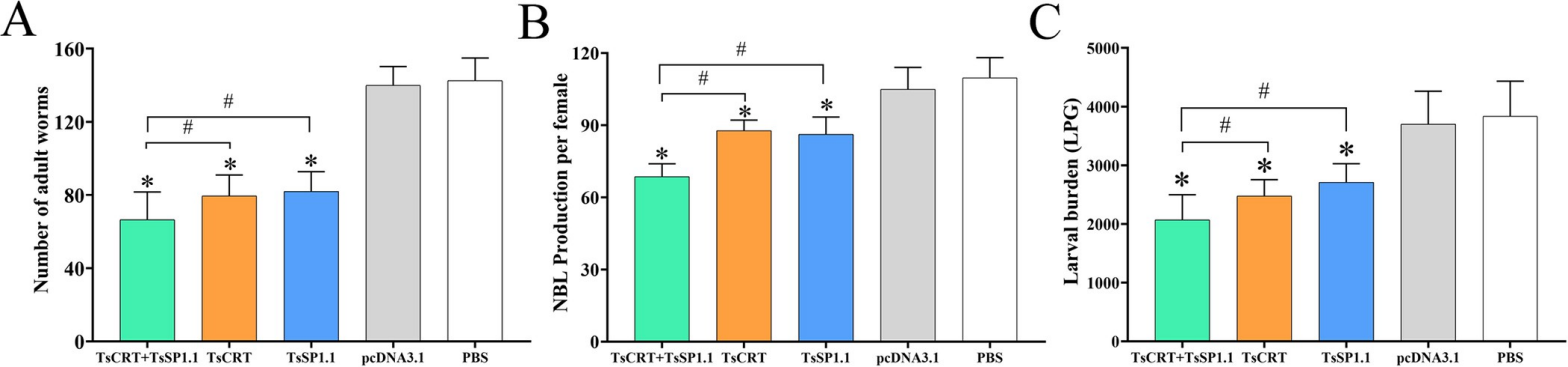
Immune protection of vaccination of mice with TsCRT +TsSP1.1, TsCRT and TsSP1.1 following 300 *T*. *spiralis* larval challenge. **A:** Intestinal adult worm burdens. **B:** The *in vitro* production of newborn larvae (NBL) deposited by each female in 72 h (n = 30). **C:** Muscle larvae burden (larvae per gram, LPG). The worm burdens are presented as mean ± SD from ten animals per group. * *P <* 0.001 compared to the PBS group. ^#^ *P <* 0.001 compared among various immunization groups.

Compared to the PBS group, the mice immunized with TsCRT+TsSP1.1, TsCRT or TsSP1.1 exhibited a 53.40, 44.28 and 42.46% reduction of intestinal AWs at 7 days following challenge with 300 *T*. *spiralis* infectious larvae (*F* = 88.442, *P <* 0.001). The intestinal adult burdens in TsCRT+TsSP1.1 group were significantly lower than single immunization group with TsCRT or TsSP1.1 DNA (*F* = 4.347, *P <* 0.001) (Fig 10A). Furthermore the *in vitro* NBL production of each female for 72 h from TsCRT+TsSP1.1 immunized mice was also distinctly lower than those of only TsCRT or rTsSP1.1 immunized group (*F* = 28.819, *P* < 0.0001) (Fig 10B). Additionally, vaccination of mice with TsCRT+TsSP1.1, TsCRT or TsSP1.1 showed a 46.05, 35.43 and 29.29% reduction of muscle larva burden at 35 dpi (*F* = 29.072, *P <* 0.001), the muscle larva burdens of mice vaccinated with TsCRT+TsSP1.1 were also obviously lower than individual TsCRT or TsSP1.1 DNA vaccination (*F* = 8.79, *P <* 0.001) (Fig 10C). But, inoculation of mice with only pcDNA3.1 did not show any evident reduction of intestinal adult and muscle larva burdens compared to the PBS group (*P* > 0.05). The results suggested that vaccination of mice with TsCRT+TsSP1.1, TsCRT or TsSP1.1 elicited a notable immune protection against *T*. *spiralis* challenge infection, reduced intestinal worm burden, hindered worm development and reduced female reproductive capacity, therefore, alleviated the muscle larva burden and *T*. *spiralis* infection in immunized mice. The results demonstrated that the protective efficacy of mixed immunization with TsCRT+TsSP1.1 was superior to individual DNA vaccine.

### ADCC killing and destroy on the NBL

**Fig 11:**
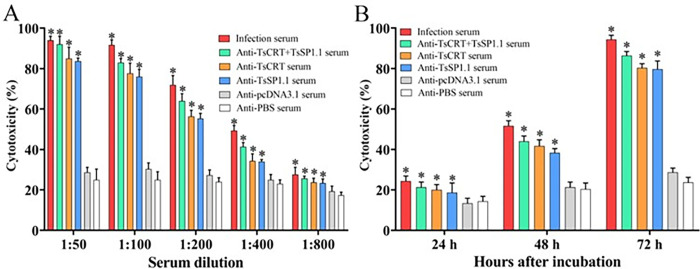
Killing effects of ADCC on the NBL. **A:** The cytotoxicity was dose-dependent of specific antibodies against TsCRT+TsSP1.1, TsCRT and TsSP1.1. **B:** The cytotoxicity had an elevating trend with prolongation of culture time. *P < 0.001 relative to sera from the PBS group.

The ADCC results showed that after culture at 37°C for 72 h, various immune sera mediated the PECs adhesion to the NBL and damage of the NBL (S2 Fig). When 1:00 dilutions of immune sera (anti-TsCRT+TsSP1.1 serum, anti-TsCRT serum and anti-TsSP1.1 serum) were supplemented into the medium and were co-cultured with the NBL as well as PECs for 72 h, the ADCC resulted in a 86.33, 80.33 and 79.67% cytotoxicity (NBL death), respectively, which were evidently higher than the sera from the pcDNA3.1 and PBS groups (*F* = 431.355, *P* < 0.001) (Fig 11A). But the cytotoxicity had no statistical differences among three groups of immune sera (*P* > 0.05). The cytotoxicity was dose-dependently related with specific antibodies against TsCRT+TsSP1.1, TsCRT and TsSP1.1 (*r*_TsCRT+TsSP1.1_ = 0.890, *r*_TsCRT_ = 0.897, *r*_TsSP1.1_ = 0.900, *P* < 0.05). Moreover, the cytotoxicity showed an elevating trend with the prolongation of culture time (*F*_24h_ = 5.81, *F*_48h_ = 68.113, *F*_72h_ = 431.355, *P* < 0.001) (Fig 11B).

### Small intestine and muscle pathological change of infected mice

**Fig 12:**
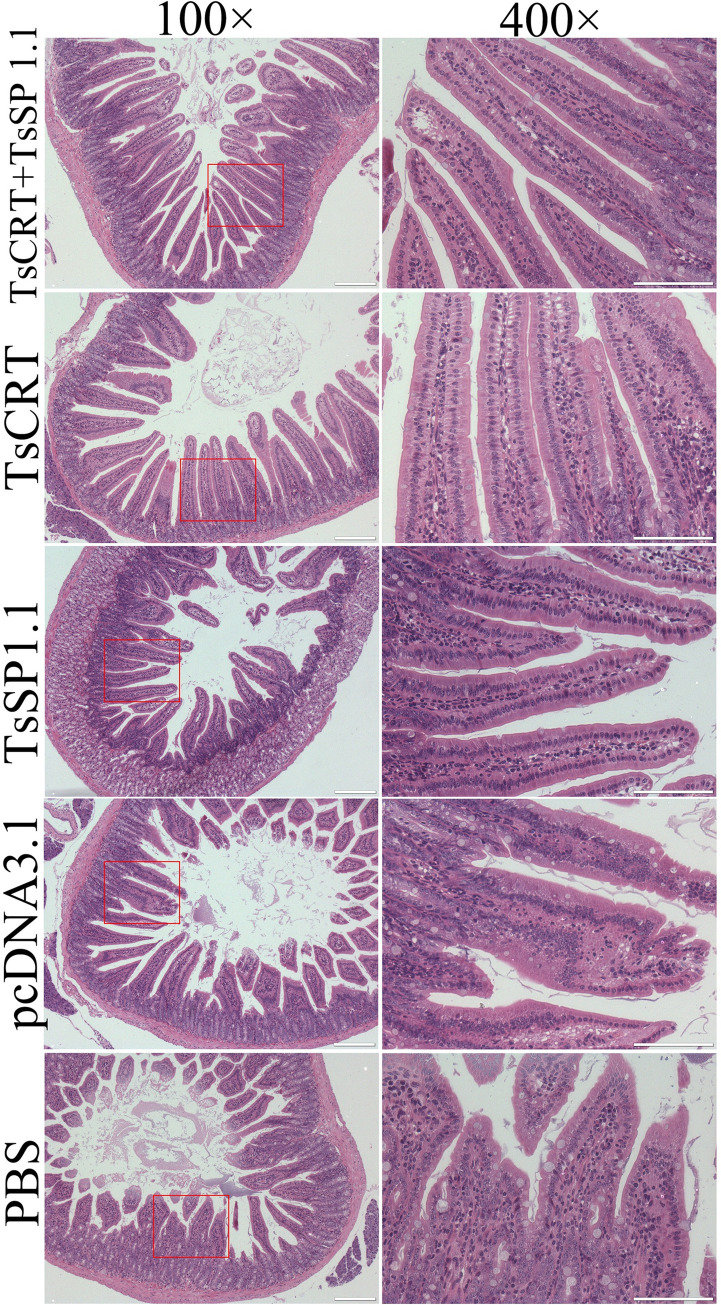
Intestinal histopathological changes in immunized mice at 7 days after challenge with 300 *T*. *spiralis* ML. Intestinal sections were stained using haematoxylin and eosin (HE) and observed on microscopy. Intestinal pathological changes from mice immunized with TsCRT+TsSP1.1, TsCRT and TsSP1.1 were significantly ameliorated. Serious intestinal mucosal inflammation, shortened and edematous intestinal villi were observed in intestinal section of the pcDNA3.1 and PBS control groups. Scale bars = 200 μm.

**Fig 13:**
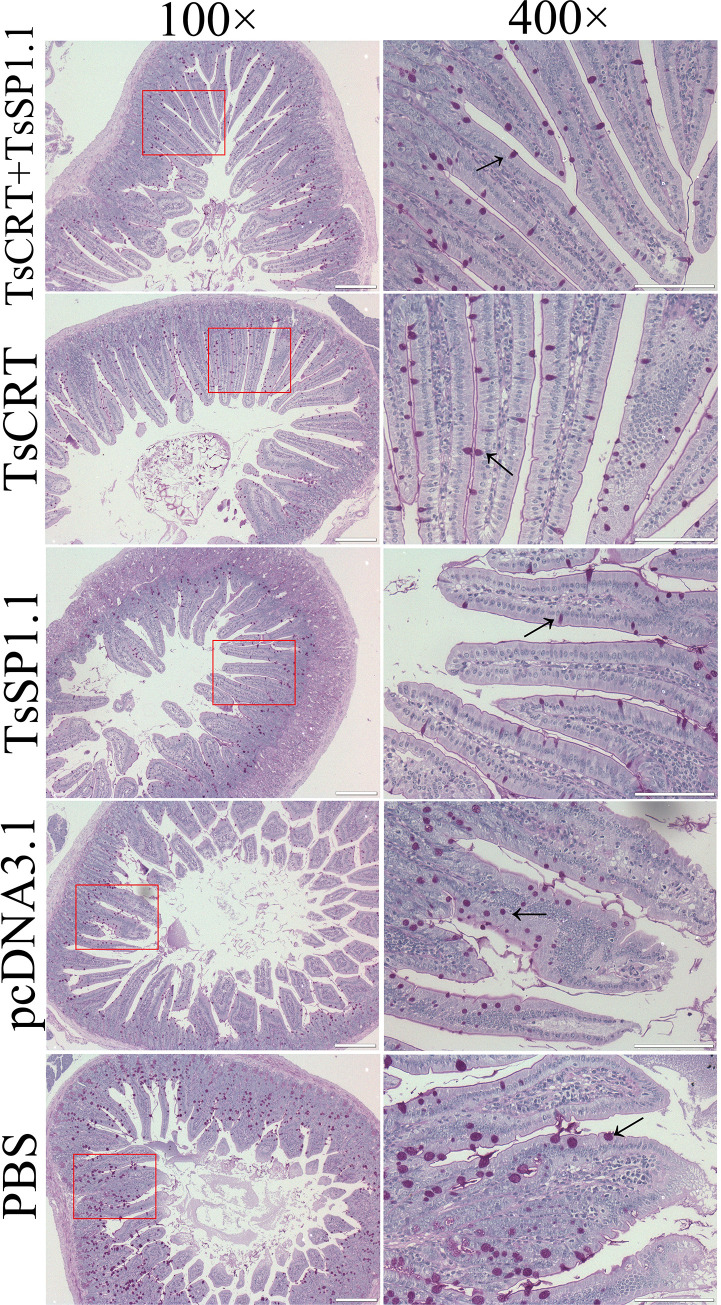
PAS staining of intestinal sections from immunized mice at 7 days after *T*. *spiralis* challenge infection. The number of goblet cells of mice immunized with TsCRT+TsSP1.1, TsCRT and TsSP1.1 was distinctly decreased compared to the pcDNA3.1 and PBS groups. Red violet goblet cells were marked with arrows. Scale bars = 200 μm.

**Fig 14:**
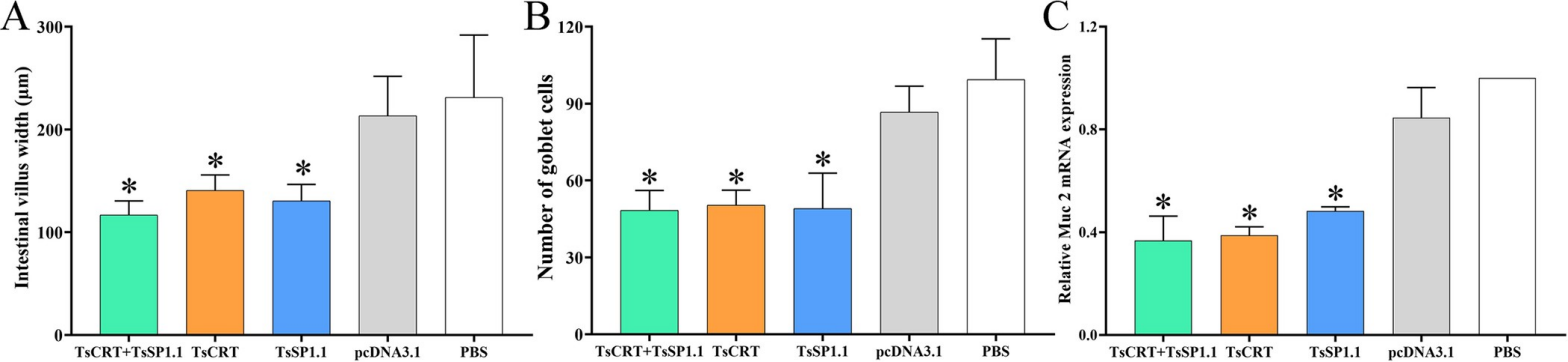
Intestinal pathological changes in immunized mice at 7 days after *T*. *spiralis* challenge. **A:** Intestinal villus width. **B:** Number of intestinal goblet cells. **C:** Relative expression level of mucin 2 mRNA. * *P* < 0.001 compared to the PBS groups.

The results of HE and PAS staining of intestinal sections revealed that at one week after challenge infection with *T*. *spiralis* larvae, mild intestinal inflammation and relative normal intestinal villi were observed in intestinal section of mice immunized with TsCRT+TsSP1.1, TsCRT and TsSP1.1 (Figs 12 and 13). Intestinal villus width of three groups of immunized mice was significantly lower than that of the pcDNA3.1 and PBS control groups (*F* = 11.605, *P <* 0.0001) (Fig 14A). Moreover, the goblet cell numbers of three groups of mice immunized TsCRT+TsSP1.1, TsCRT and TsSP1.1 were prominently less than the pcDNA3.1 and PBS groups (*F* = 13.864, *P <* 0.001) (Fig 14B). The qPCR results showed that mucin 2 transcription level of three immunized groups was also overtly lower than those of the pcDNA3.1 and PBS groups (*F* = 51.730, *P* < 0.001) (Fig 14C). The results suggested that immunization with TsCRT+TsSP1.1, TsCRT and TsSP1.1 obviously impeded larval intrusion, significantly ameliorated intestinal mucosal inflammation and reduced the mucin 2 expression.

**Fig 15:**
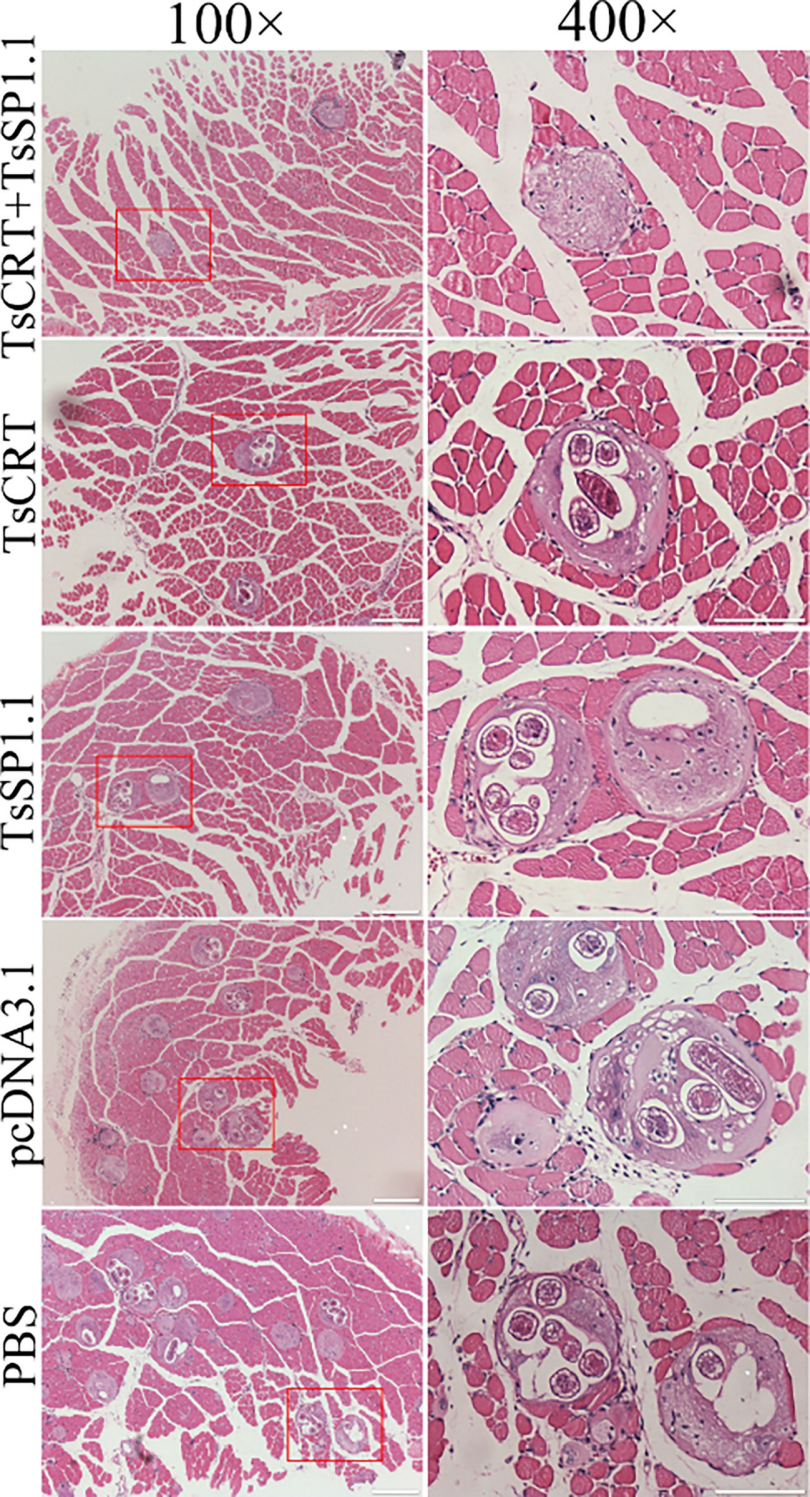
Muscle histopathological changes in immunized mice at 35 days after *T*. *spiralis* challenge. Mild inflammatory reaction and less encapsulated muscle larvae were observed in muscle section of three groups of mice immunized with TsCRT+TsSP1.1, TsCRT and TsSP1.1. Scale bars = 200 μm.

**Fig 16:**
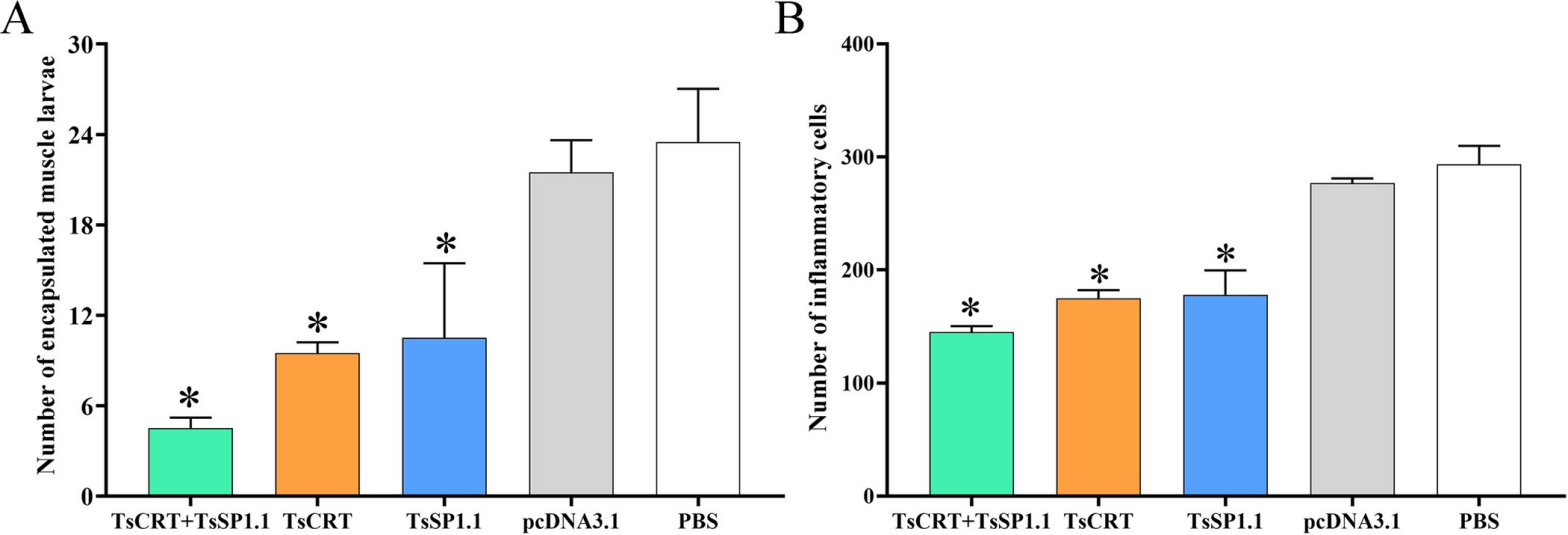
Muscle pathological changes in immunized mice at 35 days after *T*. *spiralis* challenge. **A:** The encapsulated larvae are enumerated on the muscle sections per field (100 ×). **B:** The inflammatory cells (eosinophils, neutrophils and lymphocytes) are enumerated on the muscle sections per field (400 ×). * *P* < 0.0001 compared to the PBS groups.

The results of HE staining of muscle sections of infected mice revealed that the numbers of encapsulated *T*. *spiralis* larvae of three immunized groups were distinctly lower than the PBS groups(*F =* 15.835, *P <* 0.01). Additionally, the inflammatory infiltrative cells around the encapsulated larvae of three immunized groups were also significantly reduced compared to the PBS groups (*F =* 79.709, *P <* 0.0001) (Figs 15 and 16). The number of inflammatory cells of mice immunized with TsCRT+TsSP1.1 were significantly lower than the vaccination group with only TsCRT and TsSP1.1 alone (*F =* 5.459, *P <* 0.05).

## Discussion

Trichinellosis is caused by orally ingesting infected animal meat, and enteral mucosal immune response triggered by vaccination should block larval intrusion and dislodge the intestinal nematode [21,36]. Oral vaccination is a route appropriate to elicits long gut mucosal protective immunity [53]. *S*. *typhimurium* is a gram-negative facultative anaerobic intracellular bacterium which infects orally the host, penetrates into the gut epithelial cells and enters internal organs. In murine infection, the bacteria proliferate in enteral PP, MLN and spleen [72]. Attenuated *Salmonella* is a favorable vaccine carrier that specifically delivers the antigen molecules to antigen presenting cells (APCs), and it also induces host’s immune response, and might act as a native adjuvant to enhance specific immune response evoked by target antigens [15]. Attenuated *Salmonella* as an effective oral vaccine vector has been used to induce long lasting mucosal local and systemic immune responses against intestinal nematode infection. Attenuated *S*. *typhimurium* ΔcyaSL1344 expressing *T*. *spiralis* genes (TspSP1.2, TsDNase II or TsE) has been applied to elicit gut mucosal immune response, and shown good immune protective efficacy in vaccinated animals [9,37]. Previous studies showed that there may be structural and functional differences between the proteins expressed by prokaryotic plasmids and natural proteins; the proteins expressed by DNA vaccines can fold correctly, surface-exposed antigens are also more closely related to natural proteins [73]. Therefore, Attenuated *S*. *typhimurium* was chosen as the carrier of oral DNA vaccine in this study.

*T*. *spiralis* is a multicellular intestinal and tissue parasitic nematode with a complicated life cycle, and each developmental phase has its phase-specific antigens. Immune responses elicited by vaccination using a single recombinant *T*. *spiralis* protein or DNA was not enough to protect the animals from challenge infection [16,38]. Recent studies showed that combined vaccination with pVAX1-Ts43 and pVAX1-Ts45 evoked more intense immune responses than individual DNA vaccine and resulted in higher muscle larval burden reduction [74]. Vaccination of mice with recombinant *T*. *spiralis* aminopeptidase P (rTsAPP) and cathepsin X (rTsCX) in combination produced an obvious protective immunity, demonstrated as a 68.50% muscle larva reduction [55]. Oral vaccination of mice with recombinant *L*. *plantarum* expressing Nudix hydrolase and 43 kDa proteins conferred a significant protection against *Trichinella* infection [58]. When combined vaccination using a *T*. *spiralis* serine protease (TsSP) DNA with rTsSP protein was performed, vaccinated mice showed a more worm reduction than only TsSP DNA or rTsSP alone [75]. However, *T*. *spiralis* infective larvae were not entirely eliminated from challenged animal meats, demonstrating that anti-*Trichinella* vaccines available are not sufficient to interrupt and control *Trichinella* infection [8]. Therefore, novel polyvalent vaccines against *Trichinella* intrusion, development and survival are needed to be further developed to eliminate the nematode from gut and muscles in food animals.

In this study, the fusion gene TsCRT+TsSP1.1 was designed and synthesized, the fusion gene TsCRT+TsSP1.1, individual TsCRT or TsSP1.1 was used to construct the DNA vaccines. After the 293T cells were transfected using three recombinant plasmids, transcription and expression of TsCRT+TsSP1.1, TsCRT and TsSP1.1 in transfected cells were detected by RT-PCR, IIFT and Western blot, indicating that recombinant plasmids were successfully prepared. Following oral vaccination of mice with TsCRT+TsSP1.1, TsCRT or TsSP1.1, expression of TsCRT/TsSP1.1 mRNA and protein in spleens and MLN of vaccinated animals were detected using RT-PCR and IIFT using specific anti-rTsCRT/rTsSP1.1 serum, suggesting that TsCRT and TsSP1.1 genes were expressed in murine internal organs. The results further confirmed attenuated *Salmonella* as a live vector could deliver the target DNA to gut local and systemic lymph tissues [15,53]. Oral vaccination with TsCRT+TsSP1.1, TsCRT or TsSP1.1, triggered evidently increase of anti-*Trichinella* antibodies (specific serum IgG, IgG1/IgG2a and IgA, and gut sIgA), it elicited both gut local mucosal (MLN and PP) and systemic (spleen) cellular immune response, as demonstrated by an obvious elevation of cytokines (IFN-γ and IL-4). Our results indicated that combined vaccination of mice with TsCRT+TsSP1.1 elicited prominently higher levels of specific serum antibody, mucosal sIgA and cellular response than either of single TsCRT or TsSP1.1 DNA vaccination. The concomitant Th1/Th2 responses acted a vital role against *T*. *spiralis* challenge infection [41,62]. The sIgA participates in gut mucosal defense and impedes the parasite penetration into gut epithelium [45,68]. The sIgA against surface antigens of intestinal *T*. *spiralis* stages (IIL and AW) also facilitated worm expulsion from the gut, passive transfer of naïve mice with anti-*Trichinella* IgA resulted in a 95% of protection against challenge [76]. The sIgA is Th2-dependent; especially IL-4 is the main cytokine which enhances IgA response, suggesting that high levels of IL-4 enhanced gut sIgA response [36]. Moreover, gut sIgA could reduce the female reproductive capacity of intestinal *T*. *spiralis* [9]. Our results revealed that the female fecundity of vaccinated mice with TsCRT+TsSP1.1 combination is significantly lower than only TsCRT or TsSP1.1 vaccination alone.

After being challenged, the mice vaccinated with TsCRT+TsSP1.1 produced a more obvious reduction of gut adult worms (53.40%) and muscle larva burdens (46.05%), compared to vaccination with either of individual TsCRT (44.28 and 42.46%) or TsSP1.1 DNA vaccine (35.43 and 29.29%) alone. But, oral vaccination of mice with empty pcDNA3.1 alone did not exhibit any distinct reduction of intestinal adult and muscle larva burdens compared to the PBS control group. A higher immune protection elicited by combined vaccination with TsCRT+TsSP1.1 might be related with high levels of IgG, IgA and sIgA, the cytokines IFN-γ and IL-4 [43,55]. The immune protection produced by oral vaccination with TsCRT/TsSP1.1 might be due to the combined roles of blocking larval intrusion, expelling worm from the gut, reducing female fecundity, and killing NBL by ADCC. Anti-*Trichinella* antibodies bond to the out cuticle of the IIL, and formed a cap-like immune complex in worm anterior, which physically blocked direct contact between IIL and gut epithelium, thus intercepted larval intrusion of gut mucosa and ceased the larval molting and development [65,77]. To investigate the cytotoxicity of anti-*Trichinella* antibodies, the ADCC test was conducted in this study. The results showed that anti-*Trichinella* antibodies facilitated the adhering and destruction of the macrophages to the NBL, and the cytotoxicity of ADCC was dose-dependent of anti-*Trichinella* antibodies, suggesting that anti-*Trichinella* antibodies participated in the NBL killing and damage in an ADCC mode [16,66]. Additionally, IFN-γ also played a protective act against *T*. *spiralis* infection by activating macrophages and enhancing their cytotoxic killing roles [78].

The results of PAS staining of intestinal sections at 1 week after challenge showed that intestinal villus width, goblet cell number and mucin 2 expression level of mice immunized with TsCRT+TsSP1.1, TsCRT or TsSP1.1 were remarkably lower than the empty pcDNA3.1 and PBS groups, indicating that oral vaccination of mice with TsCRT/TsSP1.1 clearly impeded larva intrusion of gut mucosa, relieved gut inflammation and alleviated the infection severity. Goblet cells are gut epithelial mucus secreting cells, which facilitate worm dislodgment from gut by secreting mucus, the number of goblet cells is closely related to the *T*. *spiralis* infection severity; evident proliferation of intestinal goblet cells and mucin secretion increase indicated serious *T*. *spiralis* infection [68,79]. Gut mucus is a crucial forefront to defense intestinal parasite infection. The mucus layer covers the surface of gut epithelia and plays a primary role in maintaining gut homeostasis and impeding parasite to invade gut epithelial cells and mucosal layer. The main component of mucus is mucin, a glycoprotein secreted by goblet cells that assembles to become a sticky and elastic gelatinous monolayer [80]. Intestinal nematode infection often results in mucus layer thickening. The elevated levels of gut mucus wrap around *T*. *spiralis* IIL and adults in the gut, limiting their activity. Gut mucus increase hinders the *T*. *spiralis* development and survival in host’s intestine [81]. Furthermore, histamine is secreted principally by mast cells, which can promote smooth muscle contraction and accelerate enteral peristalsis and worm expulsion from the gut [82]. The results revealed that oral immunization of mice with TsCRT+TsSP1.1, TsCRT and TsSP1.1 elicited histamine secretion increase at 2–6 weeks following vaccination, but histamine level had regressed to normal level at 5 weeks after challenge, suggesting that histamine is mainly involved in intestinal acute inflammation and worm expulsion at early stage of *T*. *spiralis* infection [61].

Additionally, the numbers of encapsulated larvae and inflammatory infiltrative cells around the larvae on muscle sections of three immunized groups were also significantly reduced compared to the PBS groups. The results demonstrated that oral vaccination with TsCRT+TsSP1.1, TsCRT or TsSP1.1 mitigated the larval burdens and alleviated inflammatory infiltration of muscle tissues of infected mice. It is likely because the IL-10 produced by oral vaccination reduces inflammatory responses to the ML in skeletal muscles at muscle stage of *T*. *spiralis* infection [16,83]. However, the infective ML was not completely eliminated from vaccinated infected animals. The development of anti-*Trichinella* vaccines might be an effective strategy to control *Trichinella* infection in domestic pigs and other food animals and to ensure meat food safety [84]. Therefore, to eliminate *T*. *spiralis* muscle larvae in food animals, other vaccination strategies including higher protective antigen screening, heterologous prime-boost vaccination and novel adjuvants are needed to be developed in further study [71,75].

In conclusion, oral vaccination of mice with TsCRT+TsSP1.1, TsCRT and TsSP1.1 DNA vaccines elicited a gut local mucosal sIgA response and systemic Th1/Th2 mixed response. Both TsCRT and TsSP1.1 had good immunogenicity, combined vaccination with TsCRT+TsSP1.1 induced obviously higher level of serum specific antibody, mucosal sIgA and cellular immune response than either of single TsCRT or TsSP1.1 DNA vaccination. Oral vaccination of mice with TsCRT+TsSP1.1 showed a 53.4% reduction of enteral adult worms and a 46.05% reduction of muscle larvae, provided a higher immune protection than either of individual TsCRT (44.28 and 42.46%) or TsSP1.1 DNA vaccine (35.43 and 29.29%) alone. Moreover, vaccination with TsCRT+TsSP1.1, TsCRT and TsSP1.1 also obviously ameliorated inflammation of intestinal mucosa and skeletal muscles of vaccinated mice after challenge. The results demonstrated that TsCRT and TsSP1.1 might be regarded the novel potential targets for anti-*Trichinella* vaccines. But other vaccination strategies including higher protective antigen screening, heterologous prime-boost vaccination and novel adjuvants are needed to be developed.

## Supporting information

S1 Fig Serum anti-*Trichinella* IgG titers measured by ELISA with ML soluble crude antigen. Anti-*Trichinella* IgG levels were assayed two weeks after the last immunization. The data are presented as the OD values of anti-*Trichinella* IgG level from ten vaccinated mice. Twenty five serum samples (1:100 dilutions) from normal mice were assessed as negative serum control. The cut-off values (0.341) are shown using a dotted line.
(TIF)

S2 Fig Killing effect of ADCC on the NBL. In the test, the NBL were incubated with various sera and 2 × 10^5^ mouse peritoneal exudate cells (PECs). Various immune sera mediated killing effects on NBL at different incubation times (magnification, ×400). Anti-TsCRT+TsSP1.1 serum, anti-TsCRT serum, and anti-TsSP1.1 serum mediated the PECs adhesion to the NBL and destroy of the NBL. Infection serum was used as positive control. Sera from pcDNA3.1 and PBS groups used as negative control. Scale bars = 200 μm.
(TIF)
