# Supplementary figures and images for: Oral vaccination of mice with attenuated Salmonella encoding Trichinella spiralis calreticulin and serine protease 1.1 confers protective immunity in BALB/c mice
Source: PLoS Negl Trop Dis. 2022 Nov 29;16(11):e0010929. doi: 10.1371/journal.pntd.0010929 (PMC9707759; doi:10.1371/journal.pntd.0010929)

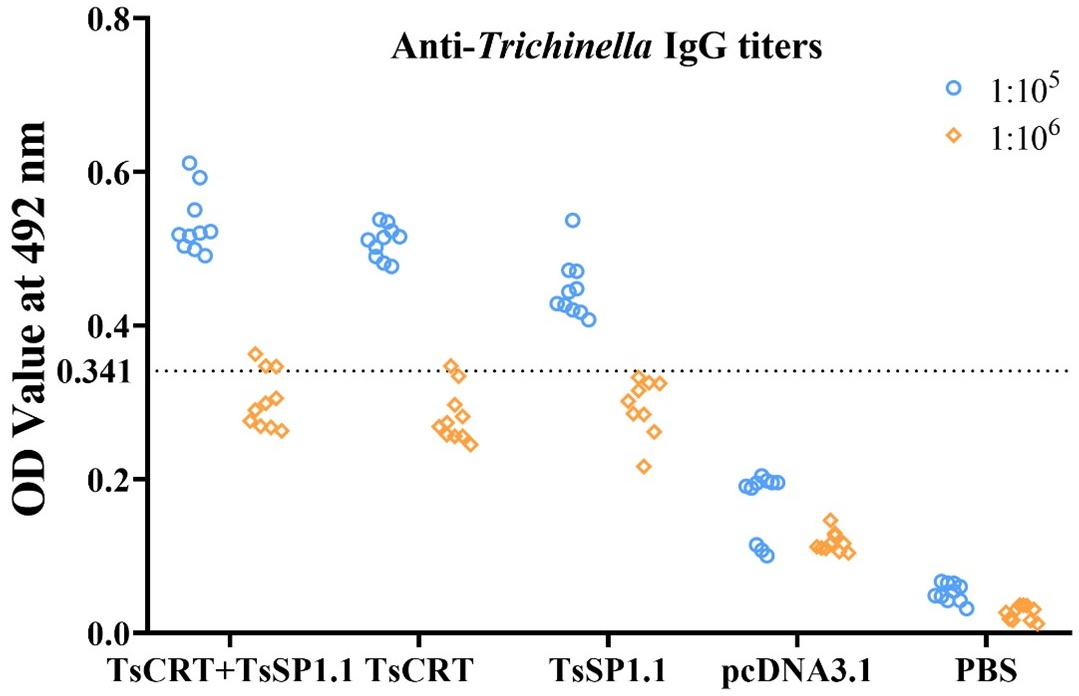

Supplement: S1 Fig — Anti-Trichinella IgG levels were assayed two weeks after the last immunization. The data are presented as the OD values of anti-Trichinella IgG level from ten vaccinated mice. Twenty five serum samples (1:100 dilutions) from normal mice were assessed as negative serum control. The cut-off values (0.341) are shown using a dotted line. (TIF) [file pntd.0010929.s001.tif]

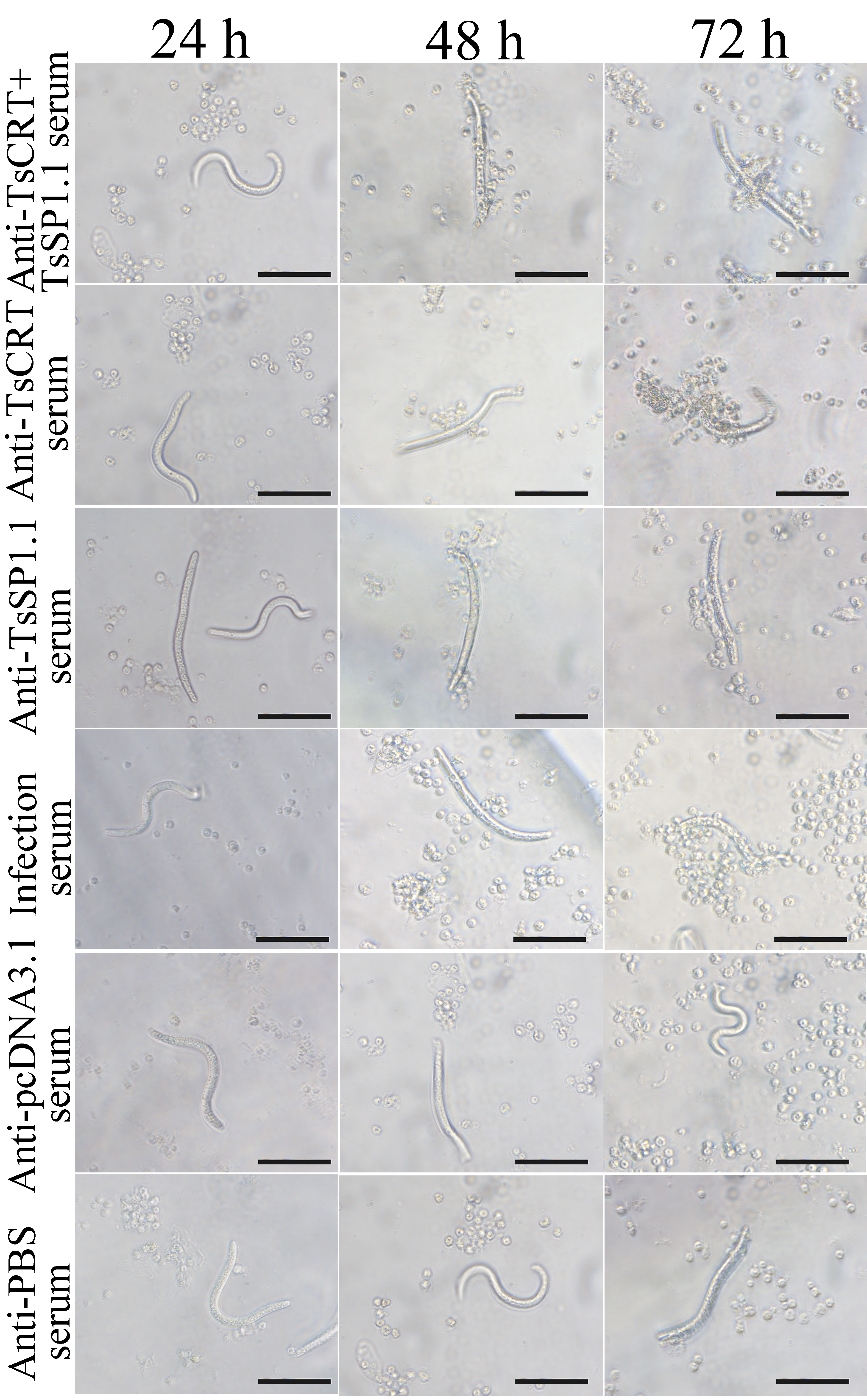

Supplement: S2 Fig — In the test, the NBL were incubated with various sera and 2 × 10^5^ mouse peritoneal exudate cells (PECs). Various immune sera mediated killing effects on NBL at different incubation times (magnification, ×400). Anti-TsCRT+TsSP1.1 serum, anti-TsCRT serum, and anti-TsSP1.1 serum mediated the PECs adhesion to the NBL and destroy of the NBL. Infection serum was used as positive control. Sera from pcDNA3.1 and PBS groups used as negative control. Scale bars = 200 μm. (TIF) [file pntd.0010929.s002.tif]
